# Context matters: assessing the impacts of genomic background and ecology on microbial biosynthetic gene cluster evolution

**DOI:** 10.1128/msystems.01538-24

**Published:** 2025-02-24

**Authors:** Rauf Salamzade, Lindsay R. Kalan

**Affiliations:** 1 Department of Medical Microbiology and Immunology, School of Medicine and Public Health, University of Wisconsin-Madison, Madison, Wisconsin, USA; 2 Microbiology Doctoral Training Program, University of Wisconsin-Madison, Madison, Wisconsin, USA; 3 M.G. DeGroote Institute for Infectious Disease Research, David Braley Center for Antibiotic Discovery, McMaster University, Hamilton, Ontario, Canada; 4 Department of Biochemistry and Biomedical Sciences, McMaster University 3710 , Hamilton, Ontario, Canada; University of Connecticut, Storrs, Connecticut, USA

**Keywords:** biosynthetic gene clusters, pangenome, *Streptomyces*, comparative genomics, secondary metabolites, natural products, evolution, ecology, population genetics, bioinformatics

## Abstract

Encoded within many microbial genomes, biosynthetic gene clusters (BGCs) underlie the synthesis of various secondary metabolites that often mediate ecologically important functions. Several studies and bioinformatics methods developed over the past decade have advanced our understanding of both microbial pangenomes and BGC evolution. In this minireview, we first highlight challenges in broad evolutionary analysis of BGCs, including delineation of BGC boundaries and clustering of BGCs across genomes. We further summarize key findings from microbial comparative genomics studies on BGC conservation across taxa and habitats and discuss the potential fitness effects of BGCs in different settings. Afterward, recent research showing the importance of genomic context on the production of secondary metabolites and the evolution of BGCs is highlighted. These studies draw parallels to recent, broader, investigations on gene-to-gene associations within microbial pangenomes. Finally, we describe mechanisms by which microbial pangenomes and BGCs evolve, ranging from the acquisition or origination of entire BGCs to micro-evolutionary trends of individual biosynthetic genes. An outlook on how expansions in the biosynthetic capabilities of some taxa might support theories that open pangenomes are the result of adaptive evolution is also discussed. We conclude with remarks about how future work leveraging longitudinal metagenomics across diverse ecosystems is likely to significantly improve our understanding on the evolution of microbial genomes and BGCs.

## INTRODUCTION

Microbial secondary metabolites are compounds produced by bacteria and fungi that are not required for their replication and unconditional survival (1, 2). While they are thus not expected to be universally essential, whereby organisms will typically survive on media meeting nutritional requirements if biosynthetic gene clusters (BGCs) are functionally impaired (3, 4), they can certainly be conditionally essential for the ecological success and survival of microbes in their natural habitats (5–9). Investigations to uncover new secondary metabolites and improve understanding of biosynthetic gene clusters have been fueled by their importance in medicine. For instance, most antibiotics used in the clinic are derived from microbial secondary metabolites (10–12). Secondary metabolites can also correspond to other natural products with uses in the fields of medicine and agriculture (2, 13, 14), function as virulence factors (15–18), and be involved in intra- or inter-species communication (19–23).

In recent times, the search for new natural products often begins with genomic prediction of BGCs (14, 24, 25). BGCs are co-located sets of genes along genomes that underlie the synthesis of secondary metabolites (26–29). The upstream use of genomics in drug discovery pipelines owes to lowering costs in sequencing over the past two decades (30, 31) and the development of bioinformatic tools and strategies to annotate BGCs (32–40). These methods range from rule-based approaches to determine indicators of genomic regions corresponding to specific types of BGCs (32–35) to generalizable approaches based on machine learning (36–38). The widespread application of these tools has revealed that while most microbes have a limited number of BGCs, the genomes of some species or genera can be rich biosynthetic reservoirs, featuring over 30 distinct BGCs per genome (41–43).

A pragmatic appeal for identifying secondary metabolites produced by BGCs rather than those with more complex pathways of biosynthesis is to simplify downstream manufacturing and production (13, 44). In particular, advances in heterologous cloning and expression of large genomic regions enable transferring the production of a metabolite to a more genetically tractable model organism (44–47). In addition, certain types of BGCs, such as those featuring polyketide synthases (PKSs) or non-ribosomal peptide synthetases (NRPSs), are known to direct the synthesis of structurally complex molecules of diverse biological functions (48). However, genomic context and complex, hierarchical mechanisms for transcriptional inhibition can often result in hurdles when attempting to determine the secondary metabolite products of BGCs under laboratory conditions (49–56).

In the last decade, several studies have advanced our understanding of the variability in the size and fluidity of microbial pangenomes—the total collection of genes found across all the genomes of a single species (57–63). One particularly exciting area of research has been to uncover relationships between genes in pangenomes by investigating whether pairs associate or dissociate with each other more often than expected (59, 64–66). In this regard, genes within a pangenome have even been compared to interacting species within a microbiome (59, 65). Comparative genomics studies have also shown that BGCs can be conserved across species or genera (67) and develop intricate relationships with other genes across genomes which can complicate secondary metabolism pathways (49, 68, 69).

In this minireview, we summarize recent advances in understanding the distribution and evolution of microbial biosynthetic gene clusters across environmental, taxonomic, and genomic contexts. Existing challenges and areas in need of further research are noted throughout.

## EVOLUTIONARY AND OPERATIONAL CLUSTERING OF BGCs

To understand the evolution of BGCs within a species, it is essential to first determine ancestrally related instances of each BGC across multiple genomes. Similar BGCs from separate genomes can be clustered according to sequence and syntenic similarity into gene cluster families (GCFs). Some approaches for delineating a GCF aim to group together operationally equivalent BGCs that produce the exact same metabolite (70–73), whereas others are more lenient and simply aim to cluster orthologous or homologous instances of BGCs together (74–77).

A challenge with grouping BGCs that produce the same metabolite is that genetic regulation, diversity in auxiliary genes, such as tailoring enzymes, and the presence of genes elsewhere in the genome, outside the BGC context, can lead to the production of chemically distinct derivatives (51, 78, 79). In addition, such approaches require manually curated data sets that link metabolites to BGCs to optimize clustering parameters, which are currently limited (71, 80). Clustering orthologous BGCs using only sequence and syntenic similarity is also challenging due to some biosynthetic genes featuring multiple domains and potentially representing a mixture of ancestral origins (8, 74, 81). This is particularly important for large, modular NRPSs and PKSs which can evolve through the exchange or gain of domains via recombination or gene conversion, respectively (8, 82, 83).

More generally, complications in GCF clustering result from the fundamental challenge of accurately inferring genomic boundaries for BGCs (36, 37, 51, 75, 84, 85), and singular regions can contain multiple associated or independent BGCs (86). This is especially problematic in BGC-rich organisms where boundaries between BGCs are unclear and difficult to resolve. In such organisms, certain classes of biosynthetic proteins further exhibit contiguous stretches of high-sequence identity and are found in higher copy count (8), causing assembly fragmentation along BGC regions. Since the vast majority of genomic assemblies on NCBI, especially those constructed from metagenomic sequencing, are not complete, there is thus an additional challenge in needing to account for BGC fragmentation when determining GCFs (71, 73, 77, 87, 88).

In recent years, a useful complement to defining GCFs has emerged. Methods have been developed to identify smaller, co-occurring subsets of genes or domains that might traverse many different BGC contexts (89, 90). These sub-clusters often underlie the synthesis of chemical functional groups or other individual features of larger molecules and compounds. A primary advantage for cataloging and assessing the presence of these sub-clusters is that they are easier to associate with chemical structures from metabolomics analysis. However, they also present an interesting opportunity to organize relationships between distinct GCFs and improve fundamental understanding on how BGCs originate and evolve. For instance, it will be particularly useful to assess how often such sub-clusters form through convergent as opposed to vertical or horizontal evolution.

## THE CONSERVATION AND FITNESS EFFECTS OF BGCs ACROSS TAXONOMIC RANKS AND HABITATS

**Fig 1:**
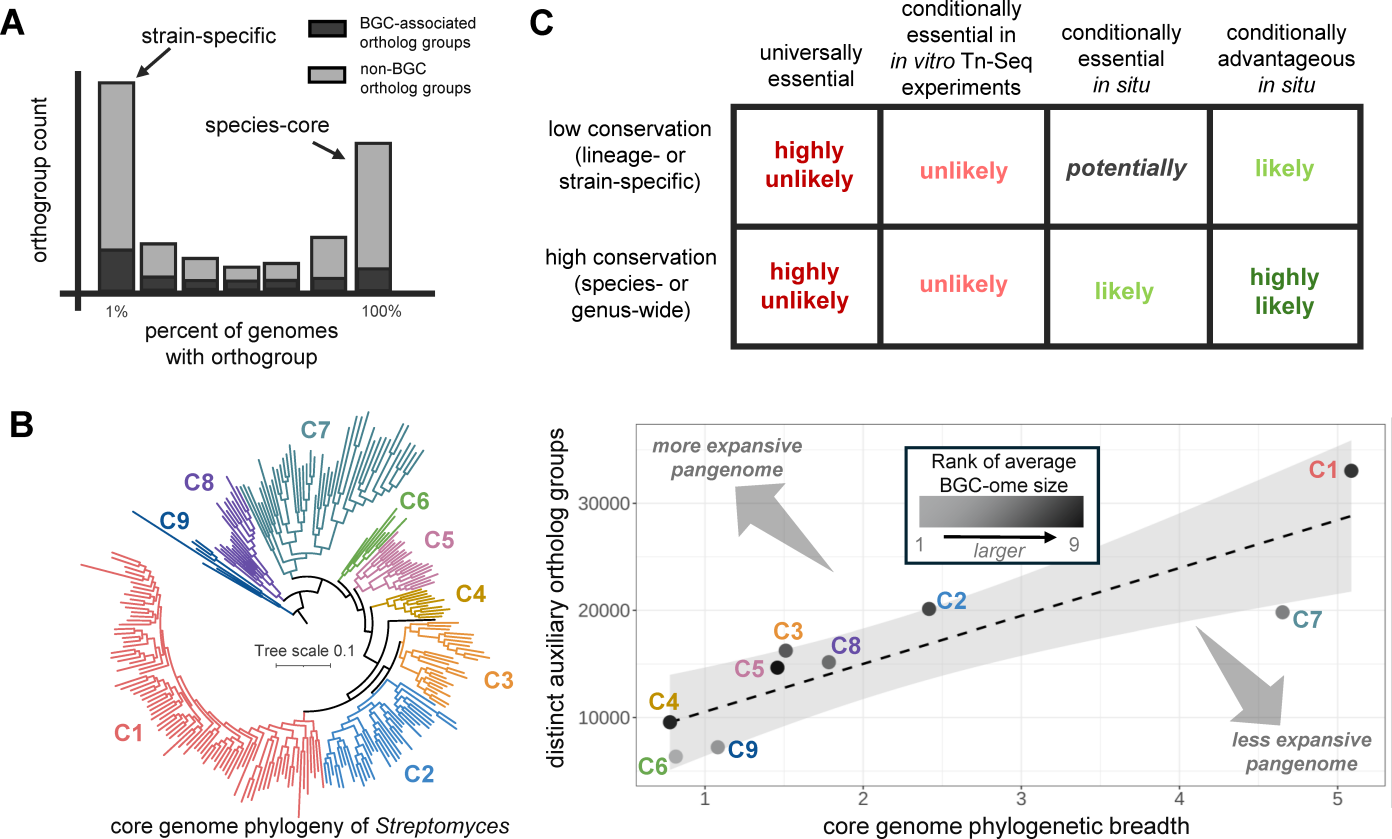
BGC conservation across pangenomes and fitness effects. (**A**) An example schematic of the frequency of ortholog groups in a typical pangenome for a bacterial species. Coloring indicates whether orthogroups were part of BGCs (dark gray) or not (light gray). (**B**) A core-genome phylogeny was constructed for 258 distinct *Streptomyces* genomes using GToTree from protein alignments for genes predetermined to be largely single-copy core for Actinomycetota. Nine monophyletic clades were manually identified, and a course, comprehensive orthology inference was performed across all genomes using OrthoFinder. The relationship between core genome phylogenetic breadth (*x*-axis) and the total number of distinct auxiliary orthogroups (*y*-axis) was visualized per clade and a linear line fit. BGCs were predicted for the 258 genomes using antiSMASH, and the average BGC-ome size—the summed length of BGC-omes—was computed for each of the nine clades. Clades above the line are regarded as having more expansive pangenomes due to the presence of a greater number of orthogroups per core-genome phylogenetic unit. Such clades also tended to have larger average BGC-ome sizes. (**C**) Current outlook on the fitness impact of BGCs based on literature and Tn-Seq studies. Details on bioinformatics (re-)analyses can be found at https://github.com/Kalan-Lab/Salamzade_Kalan_BGC_Evolution_Review (119).

Although the determination of GCFs has room for improvement, recent investigations of their distribution across species phylogenies have consistently revealed that many are lineage, species, or genus specific (15, 41, 73, 77, 91–101). One systematic analysis of the variability of GCF counts observed at different taxonomic scales provided robust support that many BGCs originate at the genus or species level (67). The specificity of some BGCs to individual genera, species, or strains is expected provided the presumption that secondary metabolites are conditionally, but not universally, important (4–7) alongside prior investigations highlighting that more than half of the genes for a species can be strain specific (57, 102–107) (Fig. 1A). Studies have also shown that biogeographic associations can be observed for microbial lineages, mobile genetic elements (MGEs), genetic traits, and metabolites (108–111). More recently, a study investigating metagenomic data sets offered broad support for the concept that many gene families are habitat specific (112). Thus, the lineage specificity of BGCs is likely related to the ecological function of their secondary metabolite products, allowing microbes to utilize endemic resources or overcome abiotic or biotic stressors associated with specific habitats (6, 68, 104, 113–118).

The genomes and pangenomes of bacterial species that inhabit a diverse range of habitats also tend to be larger than those of species that are more specialized to particular niches (62, 120). By corollary, this suggests that taxa with more open pangenomes are also likely to have larger BGC-omes—the summed length of total BGCs per genome—for fitness benefits across multiple environments. Indeed, by investigating a diverse selection of 258 genomes belonging to *Streptomyces*, which is the most heavily mined bacterial genus for natural products (121), we observe that clades with more expansive pangenomes also have larger average BGC-ome sizes (122–124) (Fig. 1B). However, future studies are clearly needed to validate these conclusions and understand the interplay between pangenome dynamics and biosynthetic potential for taxa.

In addition, while some BGCs are variably conserved, absent in some of the genomes for a taxon, others might be highly conserved across entire species or even genera (Fig. 1A). The evolution of these “core” BGCs is thus expected to be in high linkage with their genomic background (97, 125, 126) and might even suggest that they are universally essential for microbes. However, data from a recent transposon sequencing (Tn-Seq)-based study aiming to understand the relationship between gene conservation and fitness across over 30 distinct strains of *Streptococcus pneumonia*e suggest that BGCs, regardless of conservation, are typically not universally essential (127) (Fig. 1C). Assessment of the 520 essential genes identified for *S. pneumoniae* in the study, including both conserved and strain-variable genes, revealed that none overlapped with the 63 distinct key biosynthetic genes (124). In addition, we extracted fitness values for BGCs from Tn-Seq experiments for *Pseudomonas fluorescens* strain FW300-N2E3 where gene essentiality was tested *in vitro* across 125 conditions (128), ranging from growth on various carbon sources to exposure to different stressors. Only 2 of 26 key biosynthetic genes predicted in the strain’s genome were found to contribute to a growth disadvantage in at least one condition. Importantly, such Tn-Seq experiments, where thousands of distinct mutants of the same bacterial strain are pitted against each other, likely underestimate the fitness advantage offered by genes underlying public goods, such as some siderophores (129).

Furthermore, as highlighted in other recent reviews (7–9), the conservation of some BGCs across species or genera suggests that they are likely essential, or at least conditionally advantageous, beneficial but not critical for microbial fitness, under some set of conditions. Such conditions are likely linked to the specific environments that microbial taxa commonly inhabit. Indeed, compelling evolutionary and experimental support has shown that BGCs that encode for virulence factors or antimicrobials can be essential for host-colonization (15, 130–132) or combating frequently encountered microbial competitors (23, 114, 117, 133–135), respectively. Future *in vivo* and *in situ* Tn-Seq studies (136–140) could thus reveal the conditional importance of many more BGCs. Notably, longer experiments will likely be important for the proper assessment of secondary metabolite fitness effects since BGCs are typically expressed during the exponential and late growth phases of bacterial life cycles (141, 142). New bioinformatics toolkits that simplify comparative genomic analyses and emphasize examination of BGCs for focal taxonomic groups (77, 143, 144) should also aid future studies to improve understanding of the relationship between BGC conservation and fitness effect.

## BGCs ARE NOT SECLUDED UNITS WITHIN GENOMES

**Fig 2:**
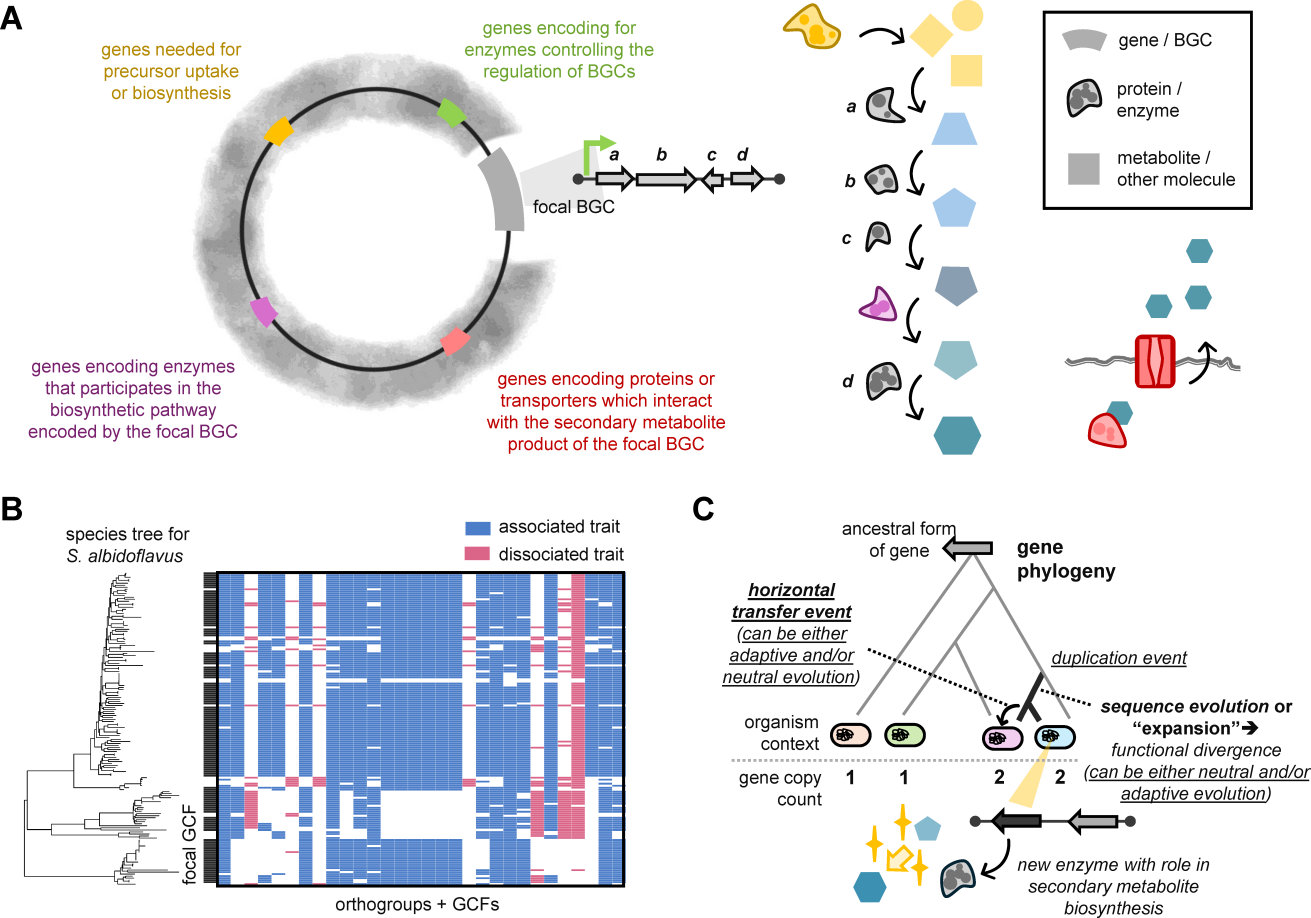
Genome-wide factors influencing secondary metabolism and evolutionary forces shaping BGCs. (**A**) A schematic showing genome-wide factors that can influence secondary metabolite biosynthesis and activity. (**B**) Orthogroups and GCFs identified using *lsa*BGC-Sociate as associated (co-occurring) or dissociated with some focal GCF from *Streptomyces albidoflavus*. (**C**) A schematic of how sequence expansion and horizontal gene transfer of genes in primary metabolism can lead to the production and spread of enzymes with new functional roles in secondary metabolite biosynthesis. Details on bioinformatics (re-)analyses can be found at https://github.com/Kalan-Lab/Salamzade_Kalan_BGC_Evolution_Review (119).

BGCs exist within larger genomic contexts, and secondary metabolites require precursor molecules (8, 145, 146). Beyond aiding fundamental evolutionary investigations of microbial pangenomes, software for performing comprehensive comparative genomics that focus on BGCs can lead to practical insights into how secondary metabolite synthesis might depend on multiple BGCs or multiple genes distributed across genomes (51, 56, 147–149) (Fig. 2A). For instance, in many *Staphylococcus* species, the *crt* BGC, which encodes for the synthesis of the carotenoid staphyloxanthin, is found co-located near the mevalonate pathway, which produces upstream precursor molecules for terpenoid biosynthesis (77, 150). In addition, attempts to synthesize staphyloxanthin in *Escherichia coli* revealed the importance of a sixth gene, *aldH*, which is not part of the five gene *crt* operon and is located elsewhere in staphylococcal genomes (49). The *aldH* encoded enzyme is responsible for catalyzing an intermediate step in staphyloxanthin biosynthesis, not merely a downstream modification, highlighting how secondary metabolite synthesis, even when seemingly restricted to a single BGC, can be fundamentally dependent and impacted by enzymes from across the genome.

Detecting genes that co-occur or are mutually exclusive to BGCs in a genome more often than would be statistically expected is thus an exciting area of methods development and research that could lead to a more holistic understanding of complex biosynthetic routes for some secondary metabolites (59, 64, 66, 151) (Fig. 2B). A recent study applied such a systematic approach to identify co-associated genes with the clinically important colibactin BGC across *E. coli* genomes (69). Broader work in pangenomics has suggested that genes can associate or dissociate from each other similar to individual species in a microbiome (59, 65). In such an analogy, biosynthetic gene clusters and operons would represent specific niches of a larger environment where significant relationships between the residing genes are likely to be enriched. While linkage is present across entire genomes in bacteria (126, 152), prior comparative genomics in *Neisseria gonorrhoeae* has found significant support that derived alleles that are within 300 kb of each other tend to exhibit greater coupling linkage (153). A systematic analysis further revealed that microbial genomes are typically segmented into 100–300 kb blocks where each block is composed of either ancient or newer genes (154). Nevertheless, resolving the boundary of biosynthetic gene clusters from the surrounding genome might be challenging when presented with limited information from genomic data alone.

Beyond genomics, other sequencing-based methods and data types, including those to quantify gene expression and fitness, can also be leveraged to improve understanding of how secondary metabolites interplay with the rest of the genome. In particular, understanding the co-regulation of BGCs and other genes is an active area of research to gain insight into the potential function of BGCs and determine which conditions they might be expressed in (51, 53, 155–161). Dissecting regulons and regulatory networks is particularly informative in BGC-rich species such as *Streptomyces*, where identifying sets of co-regulated BGCs can help researchers partition them as being related to a common cellular response for a specific ecological challenge (160). This task is challenging, however. Even in species where genomes typically contain a limited set of BGCs, secondary metabolism regulation can be complex, and a single BGC might be involved as a response to multiple environmental cues (162, 163).

In addition to standard untargeted and targeted transcriptomics, chromatin immunoprecipitation sequencing (ChIP-Seq), a technology used to determine transcription factor binding sites, has also been applied to understand the regulation of BGCs (155, 164–166). Furthermore, transposon mutagenesis and screening of arrayed mutant libraries have been used to investigate the metabolic effects and functional roles of genes within BGCs (167–169). However, the use of pooled mutant libraries and high-throughput Tn-Seq-based methods for identifying how BGCs might relate to their genomic context is perhaps underused (170–176). While there are challenges with using Tn-Seq to profile the fitness importance of BGCs, as we detail in the previous section, technological advances have enabled the generation of large data sets profiling the fitness of microbial genes across a multitude of conditions (128, 177, 178). Mining these data sets to identify co-fit genes (128), genes that exhibit similar fitness profiles across conditions, could lead to insights into the function of secondary metabolites produced by uncharacterized BGCs.

The evolution of BGCs can also depend on their genomic context. In a recent study, investigators found that two GCFs, each encoding for siderophore biosynthesis, did not co-occur in individual genomes from the genus *Salinospora* (68). This finding highlighted how BGC loss can occur when a functionally analogous BGC emerges to remove genetic redundancy and increase fitness. In contrast, another study applied informatics analysis to identify genes co-evolving with PKSs in *Streptomyces* and uncovered a discrete cluster of such genes that experimentation validated can increase the production of multiple metabolites (54). The application of methods to infer epistatic loci that exhibit signatures of co-selection within and outside of BGCs (179, 180) represents an area of research that should assist in improving understanding of how BGCs relate to their genomic context.

## THE ROLES OF ADAPTIVE AND NEUTRAL EVOLUTION ON BGCs

Inferring insight into the ecology of organisms based on signatures of adaptive evolution in genes with known function is referred to as reverse ecology (107, 181, 182). However, the extent to which microbial genomes and BGCs are sculpted by adaptive as opposed to neutral evolution is unclear (107, 183–187). In particular, complications arise because support for adaptive evolution is often contingent on ecological context, the course of evolutionary time being investigated, and sequence representation in analyses (107, 186). In this section, we discuss how both neutral and adaptive evolution can shape BGC evolution. First, we discuss how increased biosynthetic capacity might support adaptive evolution that drives pangenome expansions. Then, we summarize recent theories on the formation and evolution of BGCs, highlighting support for both adaptive and neutral evolution in shaping BGCs. Finally, we discuss current uncertainty around whether key or auxiliary biosynthetic enzymes are differentially impacted by adaptive evolution.

Beyond mutations, horizontal gene transfer (HGT) and gene sweeps that involve functional traits of potential benefit to microbial fitness and are therefore positively selected under some environmental conditions are often regarded as cases of adaptive evolution (107, 152, 188, 189). Expanding on this, McInerney and colleagues further suggested that highly open pangenomes in bacteria are also largely the result of adaptive evolution (57), but this was contradicted by other studies that suggested pangenomes can simply arise through neutral evolution (58, 103). Another perspective piece on the topic later suggested that investigations of individual genes or gene categories might lead to a better understanding of whether pangenome expansion is largely the product of adaptive as opposed to neutral evolution (190).

Provided that many secondary metabolites mediate microbe-microbe interactions, host-microbe interactions, or response to intermittent environmental challenges, it is expected that BGCs and their individual genes are more commonly under adaptive evolution relative to other gene categories (6–8, 15, 42, 114, 133, 191–196). If future research supports that pangenome expansion rates and increased biosynthetic potential are associated for certain taxa (Fig. 1B), then this might more broadly support the proposal by McInerney and colleagues (57) that expansive pangenomes can be a result of adaptive evolution. This is because the accumulation of BGCs in a taxon’s pangenome, through long-term retention of gene duplications or horizontal acquisitions, two mechanisms of biosynthetic gene gain (Fig. 2C), suggests an expansion in conditionally important gene content.

Phylogenetic analysis has shown that duplication and sequence divergence, or “expansion,” of primary metabolic genes have led to the origin of new enzymes involved in secondary metabolism (7, 8, 197–200). Evolutionary shifts in genes and enzyme function can be subtle, in particular for promiscuous enzymes that can interact with a broad range of substrates (8, 148, 201). In contrast, it can also be abrupt, through duplication events of core biosynthetic genes, such as PKSs and NRPSs leading to functional redundancy and evolutionary bifurcation. Such bifurcation can then allow microbial populations to more radically “explore” new biosynthetic pathways while mitigating the risks of losing a functional version of the BGC (202). Mechanisms of sequence evolution following duplication can extend beyond mutation. Owing to large stretches of sequence conservation and skews in nucleotide composition, sequence evolution for large and modular biosynthetic genes, such as PKSs and NRPSs, can often involve recombination and gene conversion with homologous genes from across the genome (8, 203). Additionally, the dynamic chemical matrix evolutionary hypothesis was recently formulated and posits that expanded enzymes can aggregate within genomes to form new BGCs and over time, through negative and positive selective pressures, become optimized for the production of secondary metabolites that serve specific ecological functions (8). Recent reports that genes tend to aggregate by age along microbial genomes appear to provide compelling support for this model (154). Importantly, because gene order dictates the synthesis of metabolites for some “assembly-line” BGC classes (48), duplication events of biosynthetic genes or full BGCs can also result in selection-driven changes to gene order, as suggested by another evolutionary model, the SNAP hypothesis (204).

BGCs can also be carried on MGEs (77, 205–210), such as plasmids, that are units of HGT through conjugation, transduction, transformation, or other mechanisms. In particular, *Streptomyces* and other BGC-rich actinomycetes are known to carry genes encoding for biosynthetic machinery on large mega-plasmids or the telomeric ends of linear chromosomes that can exhibit high variability in content between even closely related isolates (205, 207, 211, 212). While fitness costs associated with retaining BGCs are likely substantial for long-term retention in recipient bacteria of transfer events (9, 129, 213), BGCs that do not incur a huge influence on fitness could still be retained for a period of time (107). Other studies have suggested the impact of HGT on BGC-ome and genome evolution of actinomycetes to be less pronounced than earlier estimates (214) and reported that their BGCs largely evolve vertically (97, 125), where genetic drift might play a bigger role in shaping their evolution. The likely impact of neutral evolution on BGC sequence space in biosynthetically gifted actinomycetes is corroborated by the observation that some BGCs are only activated under specific conditions (160, 161, 215), providing ample pockets of time without strong selective pressure to preserve their functional integrity, thereby allowing them to deteriorate or to develop into new functional roles.

To assess selective pressures acting on BGCs in an actinomycetes species that is well known to evolve mostly vertically, we reinvestigated data from a study measuring evolutionary statistics for genes in *Mycobacterium tuberculosis* (216, 217). Genes within BGCs were found to have lower Tajima’s D values, a measurement of genetic diversity (218), in comparison to other genes found outside of BGCs (*P* = 0.01, one-sided Wilcoxon rank-sum test; Fig. 2D). While this suggests BGC genes are highly conserved and might be under purifying selection, significant differences between genes within and outside BGCs were no longer observed when comparing genes of a similar length. Thus, BGCs appear similarly conserved relative to other genes in the *M. tuberculosis* genome.

Importantly, BGCs often include “key” genes, responsible for synthesizing the scaffold of secondary metabolites, as well as “auxiliary” genes which might tailor and modify the secondary metabolite structures further (48, 73). For instance, in the BGC responsible for synthesizing aflatoxin, a toxin with a huge economic impact on the agricultural sector, there is one key biosynthesis gene encoding for the polyketide synthase PksA but several additional auxiliary genes. Some of the auxiliary enzymes are essential for tailoring the final structure of aflatoxin, whereas others affect the flux rate for how much of the metabolite is produced (219, 220). Population genetic and experimental studies have shown that balancing selection exists within the BGC for aflatoxin biosynthesis across *Aspergillus* species (221–223), with a recent analysis showing that in the species *Aspergillus flavu*s, greater sequence variation exists in auxiliary genes in the BGC, including those that might control flux rate for production of the toxin (224). Investigation of Tajima’s D values for genes from multiple BGCs in *A. flavus* genomes revealed that key genes underlying the synthesis of the chemical backbones of metabolites generally had lower values and are thus likely more impacted by purifying selection in comparison to auxiliary biosynthetic genes (*P* = 0.002, one-sided Wilcoxon rank-sum). However, a similar analysis in *Streptomyces albidoflavus* revealed a contrasting trend where key biosynthesis genes had higher Tajima’s values than auxiliary genes in BGCs, suggesting a higher proportion of the former being under balancing selection (*P* = 0.011, one-sided Wilcoxon rank-sum). Similar analyses across diverse taxa, BGC types, and habitats are thus needed to describe robust trends on whether BGCs more frequently evolve via changes to auxiliary or key biosynthesis genes.

## PERSPECTIVE

Microbes and evolution have generated a vast catalog of diverse BGCs and chemical metabolites, including many useful to us as natural products. The continued and urgent need for new chemical solutions for a variety of challenges we face in health and agriculture is still very much present (225). Leveraging evolutionary and ecological analysis has and can continue to prove useful in aiding the discovery of new natural products and selecting putative BGCs to experimentally characterize from an ever-increasing collection of microbial genomes (14, 42, 54, 67, 99, 200, 226, 227). Many fundamental questions around how BGCs evolve remain only partially addressed, in part due to conclusions being shaped by the scale of analyses performed, i.e., across a local population specific to a particular microbiome vs the global population for a taxon (107, 186, 224, 228).

Mining metagenomic data sets for BGCs that are divergent in sequence to those already characterized, including BGCs from difficult-to-culture or extinct bacteria, paired with advances in heterologous expression, has recently proven to be a successful approach for natural product discovery (47, 76, 229–235). Over the last decade, researchers have also begun to apply long-read sequencing technologies for metagenomics, leading to more contiguous BGC assemblies (233, 236–239) and using longitudinal metagenomics to identify signatures of adaptive evolution within individual microbiomes (77, 189, 240–244). Data sets that apply long-read sequencing for longitudinal investigations of microbiomes are likely to be generated in the future and appear promising for improving our understanding of BGC microevolutionary trends. Meta-analyses of such trends across microbiomes from different habitats could then further improve our holistic perspective on the extent to which adaptive as opposed to neutral evolution shapes microbial BGC-omes, genomes, and entire pangenomes.

Tracking microevolutionary trends might appear inconsequential since changes observable over realistic time spans for experiments are unlikely to significantly alter the functions of downstream secondary metabolites. However, improving our fundamental understanding of evolutionary rates and paths across taxonomic and environmental contexts, even over short time spans, would allow us to begin charting evolutionary landscapes and timescales for BGCs missed by examination of evolutionarily distant and ecologically unrelated instances. Practically, such research can aid the identification of which microbial taxa in specific environments are the most replete for natural products mining through extrapolation of evolutionary trends and tracking their pangenome breadth over time. These investigations can also guide the efficient synthesis of new secondary metabolites (245) and even help uncover fundamental trends and principles around how certain ecological conditions relate to increased biosynthetic diversity. For instance, future studies leveraging longitudinal metagenomics might lead to a better understanding of how microbial species and their BGCs evolve in the context of low- vs high-diversity microbiomes (246) or when exposed to intermittent “pulses” vs a consistent “press” of a stressor or selective pressure (247, 248).

## References

[1] Demain AL, Fang A. 2000. The natural functions of secondary metabolites, p 1–39. In Fiechter A (ed), History of modern biotechnology I. Springer, Berlin, Germany. 10.1007/3-540-44964-7_1 11036689

[2] Dias DA, Urban S, Roessner U. 2012. A historical overview of natural products in drug discovery. Metabolites 2:303–336. doi:10.3390/metabo2020303 24957513 PMC3901206

[3] Chao MC, Abel S, Davis BM, Waldor MK. 2016. The design and analysis of transposon insertion sequencing experiments. Nat Rev Microbiol 14:119–128. doi:10.1038/nrmicro.2015.7 26775926 PMC5099075

[4] Ioerger TR. 2022. Analysis of gene essentiality from TnSeq data using Transit. Methods Mol Biol 2377:391–421. doi:10.1007/978-1-0716-1720-5_22 34709629 PMC8941984

[5] Davies J. 2013. Specialized microbial metabolites: functions and origins. J Antibiot 66:361–364. doi:10.1038/ja.2013.61 23756686

[6] van Bergeijk DA, Terlouw BR, Medema MH, van Wezel GP. 2020. Ecology and genomics of Actinobacteria: new concepts for natural product discovery. Nat Rev Microbiol 18:546–558. doi:10.1038/s41579-020-0379-y 32483324

[7] Barona-Gómez F, Chevrette MG, Hoskisson PA. 2023. On the evolution of natural product biosynthesis. Adv Microb Physiol 83:309–349. doi:10.1016/bs.ampbs.2023.05.001 37507161

[8] Chevrette MG, Gutiérrez-García K, Selem-Mojica N, Aguilar-Martínez C, Yañez-Olvera A, Ramos-Aboites HE, Hoskisson PA, Barona-Gómez F. 2020. Evolutionary dynamics of natural product biosynthesis in bacteria. Nat Prod Rep 37:566–599. doi:10.1039/c9np00048h 31822877

[9] Jensen PR. 2016. Natural products and the gene cluster revolution. Trends Microbiol 24:968–977. doi:10.1016/j.tim.2016.07.006 27491886 PMC5123934

[10] Hutchings MI, Truman AW, Wilkinson B. 2019. Antibiotics: past, present and future. Curr Opin Microbiol 51:72–80. doi:10.1016/j.mib.2019.10.008 31733401

[11] Clardy J, Fischbach MA, Currie CR. 2009. The natural history of antibiotics. Curr Biol 19:R437–41. doi:10.1016/j.cub.2009.04.001 19515346 PMC2731226

[12] Clardy J, Fischbach MA, Walsh CT. 2006. New antibiotics from bacterial natural products. Nat Biotechnol 24:1541–1550. doi:10.1038/nbt1266 17160060

[13] Pham JV, Yilma MA, Feliz A, Majid MT, Maffetone N, Walker JR, Kim E, Cho HJ, Reynolds JM, Song MC, Park SR, Yoon YJ. 2019. A review of the microbial production of bioactive natural products and biologics. Front Microbiol 10:1404. doi:10.3389/fmicb.2019.01404 31281299 PMC6596283

[14] Ziemert N, Alanjary M, Weber T. 2016. The evolution of genome mining in microbes - a review. Nat Prod Rep 33:988–1005. doi:10.1039/c6np00025h 27272205

[15] Steenwyk JL, Mead ME, Knowles SL, Raja HA, Roberts CD, Bader O, Houbraken J, Goldman GH, Oberlies NH, Rokas A. 2020. Variation among biosynthetic gene clusters, secondary metabolite profiles, and cards of virulence across Aspergillus species. Genetics 216:481–497. doi:10.1534/genetics.120.303549 32817009 PMC7536862

[16] Lamont IL, Beare PA, Ochsner U, Vasil AI, Vasil ML. 2002. Siderophore-mediated signaling regulates virulence factor production in Pseudomonas aeruginosa. Proc Natl Acad Sci U S A 99:7072–7077. 11997446 10.1073/pnas.092016999 PMC124530

[17] Clauditz A, Resch A, Wieland K-P, Peschel A, Götz F. 2006. Staphyloxanthin plays a role in the fitness of Staphylococcus aureus and its ability to cope with oxidative stress. Infect Immun 74:4950–4953. 16861688 10.1128/IAI.00204-06 PMC1539600

[18] Rangel LI, Bolton MD. 2022. The unsung roles of microbial secondary metabolite effectors in the plant disease cacophony. Curr Opin Plant Biol 68:102233. 35679804 10.1016/j.pbi.2022.102233

[19] Linares JF, Gustafsson I, Baquero F, Martinez JL. 2006. Antibiotics as intermicrobial signaling agents instead of weapons. Proc Natl Acad Sci U S A 103:19484–19489. 17148599 10.1073/pnas.0608949103 PMC1682013

[20] Straight PD, Kolter R. 2009. Interspecies chemical communication in bacterial development. Annu Rev Microbiol 63:99–118. 19566421 10.1146/annurev.micro.091208.073248

[21] Teasdale ME, Liu J, Wallace J, Akhlaghi F, Rowley DC. 2009. Secondary metabolites produced by the marine bacterium Halobacillus salinus that inhibit quorum sensing-controlled phenotypes in gram-negative bacteria. Appl Environ Microbiol 75:567–572. doi:10.1128/AEM.00632-08 19060172 PMC2632121

[22] Calvo AM, Wilson RA, Bok JW, Keller NP. 2002. Relationship between secondary metabolism and fungal development. Microbiol Mol Biol Rev 66:447–459, doi:10.1128/MMBR.66.3.447-459.2002 12208999 PMC120793

[23] Andrić S, Rigolet A, Argüelles Arias A, Steels S, Hoff G, Balleux G, Ongena L, Höfte M, Meyer T, Ongena M. 2023. Plant-associated Bacillus mobilizes its secondary metabolites upon perception of the siderophore pyochelin produced by a Pseudomonas competitor. ISME J 17:263–275. doi:10.1038/s41396-022-01337-1 36357782 PMC9860033

[24] Bachmann BO, Van Lanen SG, Baltz RH. 2014. Microbial genome mining for accelerated natural products discovery: is a renaissance in the making? J Ind Microbiol Biotechnol 41:175–184. doi:10.1007/s10295-013-1389-9 24342967 PMC4070288

[25] Atanasov AG, Zotchev SB, Dirsch VM. 2021. International Natural Product Sciences Taskforce, Supuran CT. Nat Rev Drug Discov 20:200–216. 33510482 10.1038/s41573-020-00114-z PMC7841765

[26] Fischbach MA, Walsh CT, Clardy J. 2008. The evolution of gene collectives: How natural selection drives chemical innovation. Proc Natl Acad Sci U S A 105:4601–4608. doi:10.1073/pnas.0709132105 18216259 PMC2290807

[27] Cortes J, Haydock SF, Roberts GA, Bevitt DJ, Leadlay PF. 1990. An unusually large multifunctional polypeptide in the erythromycin-producing polyketide synthase of Saccharopolyspora erythraea. Nature New Biol 348:176–178. doi:10.1038/348176a0 2234082

[28] Donadio S, Staver MJ, McAlpine JB, Swanson SJ, Katz L. 1991. Modular organization of genes required for complex polyketide biosynthesis. Science 252:675–679. doi:10.1126/science.2024119 2024119

[29] Walsh CT, Fischbach MA. 2010. Natural products version 2.0: connecting genes to molecules. J Am Chem Soc 132:2469–2493. doi:10.1021/ja909118a 20121095 PMC2828520

[30] Land M, Hauser L, Jun S-R, Nookaew I, Leuze MR, Ahn T-H, Karpinets T, Lund O, Kora G, Wassenaar T, Poudel S, Ussery DW. 2015. Insights from 20 years of bacterial genome sequencing. Funct Integr Genomics 15:141–161. doi:10.1007/s10142-015-0433-4 25722247 PMC4361730

[31] Balloux F, Brønstad Brynildsrud O, van Dorp L, Shaw LP, Chen H, Harris KA, Wang H, Eldholm V. 2018. From theory to practice: translating whole-genome sequencing (WGS) into the clinic. Trends Microbiol 26:1035–1048. doi:10.1016/j.tim.2018.08.004 30193960 PMC6249990

[32] Weber T, Rausch C, Lopez P, Hoof I, Gaykova V, Huson DH, Wohlleben W. 2009. CLUSEAN: a computer-based framework for the automated analysis of bacterial secondary metabolite biosynthetic gene clusters. J Biotechnol 140:13–17. doi:10.1016/j.jbiotec.2009.01.007 19297688

[33] Khaldi N, Seifuddin FT, Turner G, Haft D, Nierman WC, Wolfe KH, Fedorova ND. 2010. SMURF: genomic mapping of fungal secondary metabolite clusters. Fungal Genet Biol 47:736–741. doi:10.1016/j.fgb.2010.06.003 20554054 PMC2916752

[34] Medema MH, Blin K, Cimermancic P, de Jager V, Zakrzewski P, Fischbach MA, Weber T, Takano E, Breitling R. 2011. antiSMASH: rapid identification, annotation and analysis of secondary metabolite biosynthesis gene clusters in bacterial and fungal genome sequences. Nucleic Acids Res 39:W339–W346. doi:10.1093/nar/gkr466 21672958 PMC3125804

[35] Tietz JI, Schwalen CJ, Patel PS, Maxson T, Blair PM, Tai H-C, Zakai UI, Mitchell DA. 2017. A new genome-mining tool redefines the lasso peptide biosynthetic landscape. Nat Chem Biol 13:470–478. doi:10.1038/nchembio.2319 28244986 PMC5391289

[36] Hannigan GD, Prihoda D, Palicka A, Soukup J, Klempir O, Rampula L, Durcak J, Wurst M, Kotowski J, Chang D, Wang R, Piizzi G, Temesi G, Hazuda DJ, Woelk CH, Bitton DA. 2019. A deep learning genome-mining strategy for biosynthetic gene cluster prediction. Nucleic Acids Res 47:e110. doi:10.1093/nar/gkz654 31400112 PMC6765103

[37] Carroll LM, Larralde M, Fleck JS, Ponnudurai R, Milanese A, Cappio E, Zeller G. 2021. Accurate de novo identification of biosynthetic gene clusters with GECCO. bioRxiv. doi:10.1101/2021.05.03.442509

[38] Sanchez S, Rogers JD, Rogers AB, Nassar M, McEntyre J, Welch M, Hollfelder F, Finn RD. 2023. Expansion of novel biosynthetic gene clusters from diverse environments using SanntiS. bioRxiv. doi:10.1101/2023.05.23.540769

[39] Reitz ZL, Medema MH. 2022. Genome mining strategies for metallophore discovery. Curr Opin Biotechnol 77:102757. doi:10.1016/j.copbio.2022.102757 35914390

[40] Skinnider MA, Dejong CA, Rees PN, Johnston CW, Li H, Webster ALH, Wyatt MA, Magarvey NA. 2015. Genomes to natural products PRediction Informatics for Secondary Metabolomes (PRISM). Nucleic Acids Res 43:9645–9662. doi:10.1093/nar/gkv1012 26442528 PMC4787774

[41] Belknap KC, Park CJ, Barth BM, Andam CP. 2020. Genome mining of biosynthetic and chemotherapeutic gene clusters in Streptomyces bacteria. Sci Rep 10:2003. doi:10.1038/s41598-020-58904-9 32029878 PMC7005152

[42] Chevrette MG, Carlson CM, Ortega HE, Thomas C, Ananiev GE, Barns KJ, Book AJ, Cagnazzo J, Carlos C, Flanigan W, et al.. 2019. The antimicrobial potential of Streptomyces from insect microbiomes. Nat Commun 10:516. doi:10.1038/s41467-019-08438-0 30705269 PMC6355912

[43] Chung Y-H, Kim H, Ji C-H, Je H-W, Lee D, Shim SH, Joo H-S, Kang H-S. 2021. Comparative genomics reveals a remarkable biosynthetic potential of the Streptomyces phylogenetic lineage associated with rugose-ornamented spores. mSystems 6:e00489-21. doi:10.1128/mSystems.00489-21 34427515 PMC8407293

[44] Baeshen NA, Baeshen MN, Sheikh A, Bora RS, Ahmed MMM, Ramadan HAI, Saini KS, Redwan EM. 2014. Cell factories for insulin production. Microb Cell Fact 13:141. doi:10.1186/s12934-014-0141-0 25270715 PMC4203937

[45] Harvey CJB, Tang M, Schlecht U, Horecka J, Fischer CR, Lin H-C, Li J, Naughton B, Cherry J, Miranda M, Li YF, Chu AM, Hennessy JR, Vandova GA, Inglis D, Aiyar RS, Steinmetz LM, Davis RW, Medema MH, Sattely E, Khosla C, St. Onge RP, Tang Y, Hillenmeyer ME. 2018. HEx: a heterologous expression platform for the discovery of fungal natural products. Sci Adv 4:eaar5459. doi:10.1126/sciadv.aar5459 29651464 PMC5895447

[46] Nah H-J, Pyeon H-R, Kang S-H, Choi S-S, Kim E-S. 2017. Cloning and heterologous expression of a large-sized natural product biosynthetic gene cluster in Streptomyces species. Front Microbiol 8:394. doi:10.3389/fmicb.2017.00394 28360891 PMC5350119

[47] Wang G, Zhao Z, Ke J, Engel Y, Shi Y-M, Robinson D, Bingol K, Zhang Z, Bowen B, Louie K, et al.. 2019. CRAGE enables rapid activation of biosynthetic gene clusters in undomesticated bacteria. Nat Microbiol 4:2498–2510. doi:10.1038/s41564-019-0573-8 31611640

[48] Fischbach MA, Walsh CT. 2006. Assembly-line enzymology for polyketide and nonribosomal Peptide antibiotics: logic, machinery, and mechanisms. Chem Rev 106:3468–3496. doi:10.1021/cr0503097 16895337

[49] Kim SH, Lee PC. 2012. Functional expression and extension of staphylococcal staphyloxanthin biosynthetic pathway in Escherichia coli. J Biol Chem 287:21575–21583. doi:10.1074/jbc.M112.343020 22535955 PMC3381123

[50] Kalan L, Gessner A, Thaker MN, Waglechner N, Zhu X, Szawiola A, Bechthold A, Wright GD, Zechel DL. 2013. A cryptic polyene biosynthetic gene cluster in Streptomyces calvus is expressed upon complementation with a functional bldA gene. Chem Biol 20:1214–1224. doi:10.1016/j.chembiol.2013.09.006 24120331

[51] Kwon MJ, Steiniger C, Cairns TC, Wisecaver JH, Lind AL, Pohl C, Regner C, Rokas A, Meyer V. 2021. Beyond the biosynthetic gene cluster paradigm: genome-wide coexpression networks connect clustered and unclustered transcription factors to secondary metabolic pathways. Microbiol Spectr 9:e00898-21. doi:10.1128/Spectrum.00898-21 34523946 PMC8557879

[52] Hoskisson PA, Seipke RF. 2020. Cryptic or silent? The known unknowns, unknown knowns, and unknown unknowns of secondary metabolism. MBio 11:e02642-20. doi:10.1128/mBio.02642-20 33082252 PMC7587438

[53] Mungan MD, Harbig TA, Perez NH, Edenhart S, Stegmann E, Nieselt K, Ziemert N. 2022. Secondary Metabolite Transcriptomic Pipeline (SeMa-Trap), an expression-based exploration tool for increased secondary metabolite production in bacteria. Nucleic Acids Res 50:W682–W689. doi:10.1093/nar/gkac371 35580059 PMC9252823

[54] Wang X, Chen N, Cruz-Morales P, Zhong B, Zhang Y, Wang J, Xiao Y, Fu X, Lin Y, Acharya S, Li Z, Deng H, Sun Y, Bai L, Tang X, Keasling JD, Luo X. 2024. Elucidation of genes enhancing natural product biosynthesis through co-evolution analysis. Nat Metab 6:933–946. doi:10.1038/s42255-024-01024-9 38609677

[55] Gehrke EJ, Zhang X, Pimentel-Elardo SM, Johnson AR, Rees CA, Jones SE Hindra Gehrke SS, Turvey S, Boursalie S, Hill JE, Carlson EE, Nodwell JR, Elliot MA. 2019. Silencing cryptic specialized metabolism in Streptomyces by the nucleoid-associated protein Lsr2. Elife 8:e47691. doi:10.7554/eLife.47691 31215866 PMC6584129

[56] Xue D, Older EA, Zhong Z, Shang Z, Chen N, Dittenhauser N, Hou L, Cai P, Walla MD, Dong S-H, Tang X, Chen H, Nagarkatti P, Nagarkatti M, Li Y-X, Li J. 2022. Correlational networking guides the discovery of unclustered lanthipeptide protease-encoding genes. Nat Commun 13:1647. doi:10.1038/s41467-022-29325-1 35347143 PMC8960859

[57] McInerney JO, McNally A, O’Connell MJ. 2017. Why prokaryotes have pangenomes. Nat Microbiol 2:17040. doi:10.1038/nmicrobiol.2017.40 28350002

[58] Andreani NA, Hesse E, Vos M. 2017. Prokaryote genome fluidity is dependent on effective population size. ISME J 11:1719–1721. doi:10.1038/ismej.2017.36 28362722 PMC5520154

[59] Beavan AJS, Domingo-Sananes MR, McInerney JO. 2024. Contingency, repeatability, and predictability in the evolution of a prokaryotic pangenome. Proc Natl Acad Sci U S A 121:e2304934120. doi:10.1073/pnas.2304934120 38147560 PMC10769857

[60] Douglas GM, Shapiro BJ. 2021. Genic selection within prokaryotic pangenomes. Genome Biol Evol 13:evab234. doi:10.1093/gbe/evab234 34665261 PMC8598171

[61] Douglas GM, Shapiro BJ. 2024. Pseudogenes act as a neutral reference for detecting selection in prokaryotic pangenomes. Nat Ecol Evol 8:304–314. doi:10.1038/s41559-023-02268-6 38177690

[62] Dewar AE, Hao C, Belcher LJ, Ghoul M, West SA. 2024. Bacterial lifestyle shapes pangenomes. Proc Natl Acad Sci U S A 121:e2320170121. doi:10.1073/pnas.2320170121 38743630 PMC11126918

[63] Vos M, Hesselman MC, Te Beek TA, van Passel MWJ, Eyre-Walker A. 2015. Rates of lateral gene transfer in prokaryotes: high but why? Trends Microbiol 23:598–605. doi:10.1016/j.tim.2015.07.006 26433693

[64] Whelan FJ, Rusilowicz M, McInerney JO. 2020. Coinfinder: detecting significant associations and dissociations in pangenomes. Microb Genom 6:e000338. doi:10.1099/mgen.0.000338 32100706 PMC7200068

[65] McInerney JO. 2023. Prokaryotic pangenomes act as evolving ecosystems. Mol Biol Evol 40:msac232. doi:10.1093/molbev/msac232 36288801 PMC9851318

[66] Gavriilidou A, Paulitz E, Resl C, Ziemert N, Kupczok A, Baumdicker F. 2024. Goldfinder: unraveling networks of gene co-occurrence and avoidance in bacterial pangenomes. bioRxiv. doi:10.1101/2024.04.29.591652

[67] Gavriilidou A, Kautsar SA, Zaburannyi N, Krug D, Müller R, Medema MH, Ziemert N. 2022. Compendium of specialized metabolite biosynthetic diversity encoded in bacterial genomes. Nat Microbiol 7:726–735. doi:10.1038/s41564-022-01110-2 35505244

[68] Bruns H, Crüsemann M, Letzel A-C, Alanjary M, McInerney JO, Jensen PR, Schulz S, Moore BS, Ziemert N. 2018. Function-related replacement of bacterial siderophore pathways. ISME J 12:320–329. doi:10.1038/ismej.2017.137 28809850 PMC5776446

[69] Mohite OS, Lloyd CJ, Monk JM, Weber T, Palsson BO. 2022. Pangenome analysis of Enterobacteria reveals richness of secondary metabolite gene clusters and their associated gene sets. Synth Syst Biotechnol 7:900–910. doi:10.1016/j.synbio.2022.04.011 35647330 PMC9125672

[70] Doroghazi JR, Albright JC, Goering AW, Ju K-S, Haines RR, Tchalukov KA, Labeda DP, Kelleher NL, Metcalf WW. 2014. A roadmap for natural product discovery based on large-scale genomics and metabolomics. Nat Chem Biol 10:963–968. doi:10.1038/nchembio.1659 25262415 PMC4201863

[71] Navarro-Muñoz JC, Selem-Mojica N, Mullowney MW, Kautsar SA, Tryon JH, Parkinson EI, De Los Santos ELC, Yeong M, Cruz-Morales P, Abubucker S, Roeters A, Lokhorst W, Fernandez-Guerra A, Cappelini LTD, Goering AW, Thomson RJ, Metcalf WW, Kelleher NL, Barona-Gomez F, Medema MH. 2020. A computational framework to explore large-scale biosynthetic diversity. Nat Chem Biol 16:60–68. doi:10.1038/s41589-019-0400-9 31768033 PMC6917865

[72] Kautsar SA, van der Hooft JJJ, de Ridder D, Medema MH. 2021. BiG-SLiCE: a highly scalable tool maps the diversity of 1.2 million biosynthetic gene clusters. Gigascience 10:giaa154. doi:10.1093/gigascience/giaa154 33438731 PMC7804863

[73] Ziemert N, Lechner A, Wietz M, Millán-Aguiñaga N, Chavarria KL, Jensen PR. 2014. Diversity and evolution of secondary metabolism in the marine actinomycete genus Salinispora. Proc Natl Acad Sci U S A 111:E1130–9. doi:10.1073/pnas.1324161111 24616526 PMC3970525

[74] Lin K, Zhu L, Zhang D-Y. 2006. An initial strategy for comparing proteins at the domain architecture level. Bioinformatics 22:2081–2086. doi:10.1093/bioinformatics/btl366 16837531

[75] Cimermancic P, Medema MH, Claesen J, Kurita K, Wieland Brown LC, Mavrommatis K, Pati A, Godfrey PA, Koehrsen M, Clardy J, Birren BW, Takano E, Sali A, Linington RG, Fischbach MA. 2014. Insights into secondary metabolism from a global analysis of prokaryotic biosynthetic gene clusters. Cell 158:412–421. doi:10.1016/j.cell.2014.06.034 25036635 PMC4123684

[76] Crits-Christoph A, Diamond S, Butterfield CN, Thomas BC, Banfield JF. 2018. Novel soil bacteria possess diverse genes for secondary metabolite biosynthesis. Nature 558:440–444. doi:10.1038/s41586-018-0207-y 29899444

[77] Salamzade R, Cheong JZA, Sandstrom S, Swaney MH, Stubbendieck RM, Starr NL, Currie CR, Singh AM, Kalan LR. 2023. Evolutionary investigations of the biosynthetic diversity in the skin microbiome using lsaBGC. Microb Genom 9:mgen000988. doi:10.1099/mgen.0.000988 37115189 PMC10210951

[78] Cary JW, Uka V, Han Z, Buyst D, Harris-Coward PY, Ehrlich KC, Wei Q, Bhatnagar D, Dowd PF, Martens SL, Calvo AM, Martins JC, Vanhaecke L, Coenye T, De Saeger S, Di Mavungu JD. 2015. An Aspergillus flavus secondary metabolic gene cluster containing a hybrid PKS–NRPS is necessary for synthesis of the 2-pyridones, leporins. Fungal Genet Biol 81:88–97. doi:10.1016/j.fgb.2015.05.010 26051490

[79] Qi F, Lei C, Li F, Zhang X, Wang J, Zhang W, Fan Z, Li W, Tang G-L, Xiao Y, Zhao G, Li S. 2018. Deciphering the late steps of rifamycin biosynthesis. Nat Commun 9:2342. doi:10.1038/s41467-018-04772-x 29904078 PMC6002545

[80] Terlouw BR, Blin K, Navarro-Muñoz JC, Avalon NE, Chevrette MG, Egbert S, Lee S, Meijer D, Recchia MJJ, Reitz ZL, et al.. 2023. MIBiG 3.0: a community-driven effort to annotate experimentally validated biosynthetic gene clusters. Nucleic Acids Res 51:D603–D610. doi:10.1093/nar/gkac1049 36399496 PMC9825592

[81] Persson E, Kaduk M, Forslund SK, Sonnhammer ELL. 2019. Domainoid: domain-oriented orthology inference. BMC Bioinformatics 20:523. doi:10.1186/s12859-019-3137-2 31660857 PMC6816169

[82] Nivina A, Yuet KP, Hsu J, Khosla C. 2019. Evolution and diversity of assembly-line polyketide synthases: focus review. Chem Rev 119:12524–12547. doi:10.1021/acs.chemrev.9b00525 31838842 PMC6935866

[83] Helfrich EJN, Ueoka R, Chevrette MG, Hemmerling F, Lu X, Leopold-Messer S, Minas HA, Burch AY, Lindow SE, Piel J, Medema MH. 2021. Evolution of combinatorial diversity in trans-acyltransferase polyketide synthase assembly lines across bacteria. Nat Commun 12:1422. doi:10.1038/s41467-021-21163-x 33658492 PMC7930024

[84] Andersen MR, Nielsen JB, Klitgaard A, Petersen LM, Zachariasen M, Hansen TJ, Blicher LH, Gotfredsen CH, Larsen TO, Nielsen KF, Mortensen UH. 2013. Accurate prediction of secondary metabolite gene clusters in filamentous fungi. Proc Natl Acad Sci USA 110. doi:10.1073/pnas.1205532110 PMC3538241 23248299

[85] Gilchrist CLM, Booth TJ, van Wersch B, van Grieken L, Medema MH, Chooi Y-H. 2021. Cblaster: a remote search tool for rapid identification and visualization of homologous gene clusters. Bioinform Adv 1:vbab016. doi:10.1093/bioadv/vbab016 36700093 PMC9710679

[86] Blin K, Shaw S, Steinke K, Villebro R, Ziemert N, Lee SY, Medema MH, Weber T. 2019. antiSMASH 5.0: updates to the secondary metabolite genome mining pipeline. Nucleic Acids Res 47:W81–W87. doi:10.1093/nar/gkz310 31032519 PMC6602434

[87] Klassen JL, Currie CR. 2012. Gene fragmentation in bacterial draft genomes: extent, consequences and mitigation. BMC Genomics 13:14. doi:10.1186/1471-2164-13-14 22233127 PMC3322347

[88] Seshadri R, Roux S, Huber KJ, Wu D, Yu S, Udwary D, Call L, Nayfach S, Hahnke RL, Pukall R, et al.. 2022. Expanding the genomic encyclopedia of Actinobacteria with 824 isolate reference genomes. Cell Genom 2:100213. doi:10.1016/j.xgen.2022.100213 36778052 PMC9903846

[89] Del Carratore F, Zych K, Cummings M, Takano E, Medema MH, Breitling R. 2019. Computational identification of co-evolving multi-gene modules in microbial biosynthetic gene clusters. Commun Biol 2:83. doi:10.1038/s42003-019-0333-6 30854475 PMC6395733

[90] Louwen JJR, Kautsar SA, van der Burg S, Medema MH, van der Hooft JJJ. 2023. iPRESTO: automated discovery of biosynthetic sub-clusters linked to specific natural product substructures. PLoS Comput Biol 19:e1010462. doi:10.1371/journal.pcbi.1010462 36758069 PMC9946207

[91] Duncan KR, Crüsemann M, Lechner A, Sarkar A, Li J, Ziemert N, Wang M, Bandeira N, Moore BS, Dorrestein PC, Jensen PR. 2015. Molecular networking and pattern-based genome mining improves discovery of biosynthetic gene clusters and their products from Salinispora species. Chem Biol 22:460–471. doi:10.1016/j.chembiol.2015.03.010 25865308 PMC4409930

[92] Steinke K, Mohite OS, Weber T, Kovács ÁT. 2021. Phylogenetic distribution of secondary metabolites in the Bacillus subtilis species complex. mSystems 6:e00057-21. doi:10.1128/mSystems.00057-21 33688015 PMC8546965

[93] Komaki H, Sakurai K, Hosoyama A, Kimura A, Igarashi Y, Tamura T. 2018. Diversity of nonribosomal peptide synthetase and polyketide synthase gene clusters among taxonomically close Streptomyces strains. Sci Rep 8:6888. doi:10.1038/s41598-018-24921-y 29720592 PMC5932044

[94] Chevrette MG, Handelsman J. 2021. Needles in haystacks: reevaluating old paradigms for the discovery of bacterial secondary metabolites. Nat Prod Rep 38:2083–2099. doi:10.1039/d1np00044f 34693961

[95] Drott MT, Rush TA, Satterlee TR, Giannone RJ, Abraham PE, Greco C, Venkatesh N, Skerker JM, Glass NL, Labbé JL, Milgroom MG, Keller NP. 2021. Microevolution in the pansecondary metabolome of Aspergillus flavus and its potential macroevolutionary implications for filamentous fungi. Proc Natl Acad Sci U S A 118:e2021683118. doi:10.1073/pnas.2021683118 34016748 PMC8166093

[96] Robey MT, Caesar LK, Drott MT, Keller NP, Kelleher NL. 2021. An interpreted atlas of biosynthetic gene clusters from 1,000 fungal genomes. Proc Natl Acad Sci U S A 118:e2020230118. doi:10.1073/pnas.2020230118 33941694 PMC8126772

[97] Chase AB, Sweeney D, Muskat MN, Guillén-Matus DG, Jensen PR. 2021. Vertical inheritance facilitates interspecies diversification in biosynthetic gene clusters and specialized metabolites. mBio 12:e02700-21. doi:10.1128/mBio.02700-21 34809466 PMC8609351

[98] Xia L, Miao Y, Cao A, Liu Y, Liu Z, Sun X, Xue Y, Xu Z, Xun W, Shen Q, Zhang N, Zhang R. 2022. Biosynthetic gene cluster profiling predicts the positive association between antagonism and phylogeny in Bacillus. Nat Commun 13:1023. doi:10.1038/s41467-022-28668-z 35197480 PMC8866423

[99] Chevrette MG, Currie CR. 2019. Emerging evolutionary paradigms in antibiotic discovery. J Ind Microbiol Biotechnol 46:257–271. doi:10.1007/s10295-018-2085-6 30269177

[100] Uppal S, Waterworth SC, Nick A, Vogel H, Flórez LV, Kaltenpoth M, Kwan JC. 2024. Repeated horizontal acquisition of lagriamide-producing symbionts in Lagriinae beetles. ISME J 18:wrae211. doi:10.1093/ismejo/wrae211 39441990 PMC11542224

[101] Yañez-Olvera AG, Gómez-Díaz AG, Sélem-Mojica N, Rodríguez-Orduña L, Lara-Ávila JP, Varni V, Alcoba F, Croce V, Legros T, Torres A, Torres Ruíz A, Tarrats F, Vermunt A, Looije T, Cibrian-Jaramillo A, Valenzuela M, Siri MI, Barona-Gomez F. 2024. A host shift as the origin of tomato bacterial canker caused by Clavibacter michiganensis. Microb Genom 10:001309. doi:10.1099/mgen.0.001309 39471242 PMC11521342

[102] Welch RA, Burland V, Plunkett G III, Redford P, Roesch P, Rasko D, Buckles EL, Liou S-R, Boutin A, Hackett J, Stroud D, Mayhew GF, Rose DJ, Zhou S, Schwartz DC, Perna NT, Mobley HLT, Donnenberg MS, Blattner FR. 2002. Extensive mosaic structure revealed by the complete genome sequence of uropathogenic Escherichia coli. Proc Natl Acad Sci U S A 99:17020–17024. doi:10.1073/pnas.252529799 12471157 PMC139262

[103] Haegeman B, Weitz JS. 2012. A neutral theory of genome evolution and the frequency distribution of genes. BMC Genomics 13:196. doi:10.1186/1471-2164-13-196 22613814 PMC3386021

[104] Cordero OX, Polz MF. 2014. Explaining microbial genomic diversity in light of evolutionary ecology. Nat Rev Microbiol 12:263–273. doi:10.1038/nrmicro3218 24590245

[105] Colquhoun RM, Hall MB, Lima L, Roberts LW, Malone KM, Hunt M, Letcher B, Hawkey J, George S, Pankhurst L, Iqbal Z. 2021. Pandora: nucleotide-resolution bacterial pan-genomics with reference graphs. Genome Biol 22:267. doi:10.1186/s13059-021-02473-1 34521456 PMC8442373

[106] Tonkin-Hill G, MacAlasdair N, Ruis C, Weimann A, Horesh G, Lees JA, Gladstone RA, Lo S, Beaudoin C, Floto RA, Frost SDW, Corander J, Bentley SD, Parkhill J. 2020. Producing polished prokaryotic pangenomes with the Panaroo pipeline. Genome Biol 21:180. doi:10.1186/s13059-020-02090-4 32698896 PMC7376924

[107] Arnold BJ, Huang I-T, Hanage WP. 2022. Horizontal gene transfer and adaptive evolution in bacteria. Nat Rev Microbiol 20:206–218. doi:10.1038/s41579-021-00650-4 34773098

[108] Green JL, Bohannan BJM, Whitaker RJ. 2008. Microbial biogeography: from taxonomy to traits. Science 320:1039–1043. doi:10.1126/science.1153475 18497288

[109] Martiny JBH, Bohannan BJM, Brown JH, Colwell RK, Fuhrman JA, Green JL, Horner-Devine MC, Kane M, Krumins JA, Kuske CR, Morin PJ, Naeem S, Ovreås L, Reysenbach A-L, Smith VH, Staley JT. 2006. Microbial biogeography: putting microorganisms on the map. Nat Rev Microbiol 4:102–112. doi:10.1038/nrmicro1341 16415926

[110] Chase AB, Bogdanov A, Demko AM, Jensen PR. 2023. Biogeographic patterns of biosynthetic potential and specialized metabolites in marine sediments. ISME J 17:976–983. doi:10.1038/s41396-023-01410-3 37061583 PMC10284892

[111] Salamzade R, Manson AL, Walker BJ, Brennan-Krohn T, Worby CJ, Ma P, He LL, Shea TP, Qu J, Chapman SB, et al.. 2022. Inter-species geographic signatures for tracing horizontal gene transfer and long-term persistence of carbapenem resistance. Genome Med 14:37. doi:10.1186/s13073-022-01040-y 35379360 PMC8981930

[112] Coelho LP, Alves R, Del Río ÁR, Myers PN, Cantalapiedra CP, Giner-Lamia J, Schmidt TS, Mende DR, Orakov A, Letunic I, Hildebrand F, Van Rossum T, Forslund SK, Khedkar S, Maistrenko OM, Pan S, Jia L, Ferretti P, Sunagawa S, Zhao X-M, Nielsen HB, Huerta-Cepas J, Bork P. 2022. Towards the biogeography of prokaryotic genes. Nature 601:252–256. doi:10.1038/s41586-021-04233-4 34912116 PMC7613196

[113] Crits-Christoph A, Bhattacharya N, Olm MR, Song YS, Banfield JF. 2021. Transporter genes in biosynthetic gene clusters predict metabolite characteristics and siderophore activity. Genome Res 31:239–250. doi:10.1101/gr.268169.120 33361114 PMC7849407

[114] Claesen J, Spagnolo JB, Ramos SF, Kurita KL, Byrd AL, Aksenov AA, Melnik AV, Wong WR, Wang S, Hernandez RD, Donia MS, Dorrestein PC, Kong HH, Segre JA, Linington RG, Fischbach MA, Lemon KP. 2020. A Cutibacterium acnes antibiotic modulates human skin microbiota composition in hair follicles. Sci Transl Med 12:eaay5445. doi:10.1126/scitranslmed.aay5445 33208503 PMC8478231

[115] Chevrette MG, Thomas CS, Hurley A, Rosario-Meléndez N, Sankaran K, Tu Y, Hall A, Magesh S, Handelsman J. 2022. Microbiome composition modulates secondary metabolism in a multispecies bacterial community. Proc Natl Acad Sci U S A 119:e2212930119. doi:10.1073/pnas.2212930119 36215464 PMC9586298

[116] Bosak T, Losick RM, Pearson A. 2008. A polycyclic terpenoid that alleviates oxidative stress. Proc Natl Acad Sci U S A 105:6725–6729. doi:10.1073/pnas.0800199105 18436644 PMC2373358

[117] Zipperer A, Konnerth MC, Laux C, Berscheid A, Janek D, Weidenmaier C, Burian M, Schilling NA, Slavetinsky C, Marschal M, Willmann M, Kalbacher H, Schittek B, Brötz-Oesterhelt H, Grond S, Peschel A, Krismer B. 2016. Human commensals producing a novel antibiotic impair pathogen colonization. Nature 535:511–516. doi:10.1038/nature18634 27466123

[118] Kraemer SM, Duckworth OW, Harrington JM, Schenkeveld WDC. 2015. Metallophores and trace metal biogeochemistry. Aquat Geochem 21:159–195. doi:10.1007/s10498-014-9246-7

[119] Salamzade R. 2025. Kalan-Lab/Salamzade_Kalan_BGC_Evolution_Review: v1.0. Zenodo. Available from: 10.5281/ZENODO.14629824

[120] Barberán A, Ramirez KS, Leff JW, Bradford MA, Wall DH, Fierer N. 2014. Why are some microbes more ubiquitous than others? Predicting the habitat breadth of soil bacteria. Ecol Lett 17:794–802. doi:10.1111/ele.12282 24751288

[121] Alam K, Mazumder A, Sikdar S, Zhao Y-M, Hao J, Song C, Wang Y, Sarkar R, Islam S, Zhang Y, Li A. 2022. Streptomyces: the biofactory of secondary metabolites. Front Microbiol 13:968053. doi:10.3389/fmicb.2022.968053 36246257 PMC9558229

[122] Emms DM, Kelly S. 2019. OrthoFinder: phylogenetic orthology inference for comparative genomics. Genome Biol 20:238. doi:10.1186/s13059-019-1832-y 31727128 PMC6857279

[123] Salamzade R, Kalan LR. 2023. skDER: microbial genome dereplication approaches for comparative and metagenomic applications. bioRxiv. doi:10.1101/2023.09.27.559801

[124] Blin K, Shaw S, Augustijn HE, Reitz ZL, Biermann F, Alanjary M, Fetter A, Terlouw BR, Metcalf WW, Helfrich EJN, van Wezel GP, Medema MH, Weber T. 2023. antiSMASH 7.0: new and improved predictions for detection, regulation, chemical structures and visualisation. Nucleic Acids Res 51:W46–W50. doi:10.1093/nar/gkad344 37140036 PMC10320115

[125] McDonald BR, Currie CR. 2017. Lateral gene transfer dynamics in the ancient bacterial genus Streptomyces. mBio 8:e00644-17. doi:10.1128/mBio.00644-17 28588130 PMC5472806

[126] Earle SG, Wu C-H, Charlesworth J, Stoesser N, Gordon NC, Walker TM, Spencer CCA, Iqbal Z, Clifton DA, Hopkins KL, Woodford N, Smith EG, Ismail N, Llewelyn MJ, Peto TE, Crook DW, McVean G, Walker AS, Wilson DJ. 2016. Identifying lineage effects when controlling for population structure improves power in bacterial association studies. Nat Microbiol 1:16041. doi:10.1038/nmicrobiol.2016.41 27572646 PMC5049680

[127] Rosconi F, Rudmann E, Li J, Surujon D, Anthony J, Frank M, Jones DS, Rock C, Rosch JW, Johnston CD, van Opijnen T. 2022. A bacterial pan-genome makes gene essentiality strain-dependent and evolvable. Nat Microbiol 7:1580–1592. doi:10.1038/s41564-022-01208-7 36097170 PMC9519441

[128] Price MN, Wetmore KM, Waters RJ, Callaghan M, Ray J, Liu H, Kuehl JV, Melnyk RA, Lamson JS, Suh Y, Carlson HK, Esquivel Z, Sadeeshkumar H, Chakraborty R, Zane GM, Rubin BE, Wall JD, Visel A, Bristow J, Blow MJ, Arkin AP, Deutschbauer AM. 2018. Mutant phenotypes for thousands of bacterial genes of unknown function. Nature 557:503–509. doi:10.1038/s41586-018-0124-0 29769716

[129] Cordero OX, Ventouras L-A, DeLong EF, Polz MF. 2012. Public good dynamics drive evolution of iron acquisition strategies in natural bacterioplankton populations. Proc Natl Acad Sci U S A 109:20059–20064. doi:10.1073/pnas.1213344109 23169633 PMC3523850

[130] Rigottier-Gois L, Madec C, Navickas A, Matos RC, Akary-Lepage E, Mistou M-Y, Serror P. 2015. The surface rhamnopolysaccharide epa of Enterococcus faecalis is a key determinant of intestinal colonization. J Infect Dis 211:62–71. doi:10.1093/infdis/jiu402 25035517

[131] Zimmermann M, Fischbach MA. 2010. A family of pyrazinone natural products from a conserved nonribosomal peptide synthetase in Staphylococcus aureus. Chem Biol 17:925–930. doi:10.1016/j.chembiol.2010.08.006 20851341

[132] Buckling A, Harrison F, Vos M, Brockhurst MA, Gardner A, West SA, Griffin A. 2007. Siderophore-mediated cooperation and virulence in Pseudomonas aeruginosa. FEMS Microbiol Ecol 62:135–141. doi:10.1111/j.1574-6941.2007.00388.x 17919300

[133] Currie CR, Scott JA, Summerbell RC, Malloch D. 1999. Fungus-growing ants use antibiotic-producing bacteria to control garden parasites. Nature 398:701–704. doi:10.1038/19519

[134] van der Meij A, Worsley SF, Hutchings MI, van Wezel GP. 2017. Chemical ecology of antibiotic production by actinomycetes. FEMS Microbiol Rev 41:392–416. doi:10.1093/femsre/fux005 28521336

[135] Heine D, Holmes NA, Worsley SF, Santos ACA, Innocent TM, Scherlach K, Patrick EH, Yu DW, Murrell JC, Vieria PC, Boomsma JJ, Hertweck C, Hutchings MI, Wilkinson B. 2018. Chemical warfare between leafcutter ant symbionts and a co-evolved pathogen. Nat Commun 9:2208. doi:10.1038/s41467-018-04520-1 29880868 PMC5992151

[136] Goodman AL, McNulty NP, Zhao Y, Leip D, Mitra RD, Lozupone CA, Knight R, Gordon JI. 2009. Identifying genetic determinants needed to establish a human gut symbiont in its habitat. Cell Host Microbe 6:279–289. doi:10.1016/j.chom.2009.08.003 19748469 PMC2895552

[137] Hubbard TP, Chao MC, Abel S, Blondel CJ, Abel Zur Wiesch P, Zhou X, Davis BM, Waldor MK. 2016. Genetic analysis of Vibrio parahaemolyticus intestinal colonization. Proc Natl Acad Sci U S A 113:6283–6288. doi:10.1073/pnas.1601718113 27185914 PMC4896720

[138] Morinière L, Mirabel L, Gueguen E, Bertolla F. 2022. Comprehensive overview of the genes and functions required for lettuce infection by the hemibiotrophic phytopathogen Xanthomonas hortorum pv. vitians. mSystems 7:e01290-21. doi:10.1128/msystems.01290-21 35311560 PMC9040725

[139] Torres M, Paszti S, Eberl L. 2024. Shedding light on bacteria-host interactions with the aid of TnSeq approaches. mBio 15:e00390-24. doi:10.1128/mbio.00390-24 38722161 PMC11237515

[140] Cole BJ, Feltcher ME, Waters RJ, Wetmore KM, Mucyn TS, Ryan EM, Wang G, Ul-Hasan S, McDonald M, Yoshikuni Y, Malmstrom RR, Deutschbauer AM, Dangl JL, Visel A. 2017. Genome-wide identification of bacterial plant colonization genes. PLoS Biol 15:e2002860. doi:10.1371/journal.pbio.2002860 28938018 PMC5627942

[141] Bibb MJ. 2005. Regulation of secondary metabolism in streptomycetes. Curr Opin Microbiol 8:208–215. doi:10.1016/j.mib.2005.02.016 15802254

[142] Amos GCA, Awakawa T, Tuttle RN, Letzel A-C, Kim MC, Kudo Y, Fenical W, S. Moore B, Jensen PR. 2017. Comparative transcriptomics as a guide to natural product discovery and biosynthetic gene cluster functionality. Proc Natl Acad Sci USA 114:E11121–E11130. doi:10.1073/pnas.1714381115 29229817 PMC5748202

[143] Alanjary M, Kronmiller B, Adamek M, Blin K, Weber T, Huson D, Philmus B, Ziemert N. 2017. The Antibiotic Resistant Target Seeker (ARTS), an exploration engine for antibiotic cluster prioritization and novel drug target discovery. Nucleic Acids Res 45:W42–W48. doi:10.1093/nar/gkx360 28472505 PMC5570205

[144] Nuhamunada M, Mohite OS, Phaneuf PV, Palsson BO, Weber T. 2024. BGCFlow: systematic pangenome workflow for the analysis of biosynthetic gene clusters across large genomic datasets. Nucleic Acids Res 52:5478–5495. doi:10.1093/nar/gkae314 38686794 PMC11162802

[145] Seyedsayamdost MR. 2019. Toward a global picture of bacterial secondary metabolism. J Ind Microbiol Biotechnol 46:301–311. doi:10.1007/s10295-019-02136-y 30684124 PMC6779422

[146] Kountz DJ, Balskus EP. 2021. Leveraging microbial genomes and genomic context for chemical discovery. Acc Chem Res 54:2788–2797. doi:10.1021/acs.accounts.1c00100 34087065 PMC8264950

[147] Yin M, Lu T, Zhao L-X, Chen Y, Huang S-X, Lohman JR, Xu L-H, Jiang C-L, Shen B. 2011. The missing C-17 O-methyltransferase in geldanamycin biosynthesis. Org Lett 13:3726–3729. doi:10.1021/ol201383w 21682254

[148] Wu C, Medema MH, Läkamp RM, Zhang L, Dorrestein PC, Choi YH, van Wezel GP. 2016. Leucanicidin and endophenasides result from methyl-rhamnosylation by the same tailoring enzymes in Kitasatospora sp. MBT66. ACS Chem Biol 11:478–490. doi:10.1021/acschembio.5b00801 26675041

[149] Tang M-C, Lin H-C, Li D, Zou Y, Li J, Xu W, Cacho RA, Hillenmeyer ME, Garg NK, Tang Y. 2015. Discovery of unclustered fungal indole diterpene biosynthetic pathways through combinatorial pathway reassembly in engineered yeast. J Am Chem Soc 137:13724–13727. doi:10.1021/jacs.5b06108 26469304 PMC4732561

[150] Pelz A, Wieland K-P, Putzbach K, Hentschel P, Albert K, Götz F. 2005. Structure and biosynthesis of staphyloxanthin from Staphylococcus aureus. J Biol Chem 280:32493–32498. doi:10.1074/jbc.M505070200 16020541

[151] Salamzade R, Kalan LR 2024. lsaBGC-Pan. https://zenodo.org/records/13182411.

[152] Shapiro BJ. 2016. How clonal are bacteria over time? Curr Opin Microbiol 31:116–123. doi:10.1016/j.mib.2016.03.013 27057964

[153] Arnold B, Sohail M, Wadsworth C, Corander J, Hanage WP, Sunyaev S, Grad YH. 2020. Fine-scale haplotype structure reveals strong signatures of positive selection in a recombining bacterial pathogen. Mol Biol Evol 37:417–428. doi:10.1093/molbev/msz225 31589312 PMC6993868

[154] Wolf YI, Schurov IV, Makarova KS, Katsnelson MI, Koonin EV. 2024. Long range segmentation of prokaryotic genomes by gene age and functionality. Nucleic Acids Res 52:11045–11059. doi:10.1093/nar/gkae745 39193895 PMC11472176

[155] Lee N, Hwang S, Kim W, Lee Y, Kim JH, Cho S, Kim HU, Yoon YJ, Oh M-K, Palsson BO, Cho B-K. 2021. Systems and synthetic biology to elucidate secondary metabolite biosynthetic gene clusters encoded in Streptomyces genomes. Nat Prod Rep 38:1330–1361. doi:10.1039/d0np00071j 33393961

[156] Augustijn HE, Reitz ZL, Zhang L, Boot JA, Elsayed SS, Challis GL, Medema MH, van Wezel GP. 2024. Prediction of gene cluster function based on transcriptional regulatory networks uncovers a novel locus required for desferrioxamine B biosynthesis. bioRxiv. doi:10.1101/2024.06.10.598258 PMC12161575 40504771

[157] Yan Q, Philmus B, Chang JH, Loper JE. 2017. Novel mechanism of metabolic co-regulation coordinates the biosynthesis of secondary metabolites in Pseudomonas protegens. Elife 6:e22835. doi:10.7554/eLife.22835 28262092 PMC5395296

[158] Bilyk B, Kim S, Fazal A, Baker TA, Seipke RF. 2020. Regulation of antimycin biosynthesis is controlled by the ClpXP protease. mSphere 5:e00144-20. doi:10.1128/mSphere.00144-20 32269155 PMC7142297

[159] McLean TC, Wilkinson B, Hutchings MI, Devine R. 2019. Dissolution of the disparate: co-ordinate regulation in antibiotic biosynthesis. Antibiotics (Basel) 8:83. doi:10.3390/antibiotics8020083 31216724 PMC6627628

[160] Augustijn HE, Roseboom AM, Medema MH, van Wezel GP. 2024. Harnessing regulatory networks in Actinobacteria for natural product discovery. J Ind Microbiol Biotechnol 51:kuae011. doi:10.1093/jimb/kuae011 38569653 PMC10996143

[161] Yu J-H, Keller N. 2005. Regulation of secondary metabolism in filamentous fungi. Annu Rev Phytopathol 43:437–458. doi:10.1146/annurev.phyto.43.040204.140214 16078891

[162] Pannu MK, Hudman DA, Sargentini NJ, Singh VK. 2019. Role of SigB and staphyloxanthin in radiation survival of Staphylococcus aureus. Curr Microbiol 76:70–77. doi:10.1007/s00284-018-1586-x 30353215

[163] Hall JW, Yang J, Guo H, Ji Y. 2017. The Staphylococcus aureus AirSR two-component system mediates reactive oxygen species resistance via transcriptional regulation of staphyloxanthin production. Infect Immun 85:e00838-16. doi:10.1128/IAI.00838-16 27872240 PMC5278164

[164] Wiemann P, Sieber CMK, von Bargen KW, Studt L, Niehaus E-M, Espino JJ, Huß K, Michielse CB, Albermann S, Wagner D, et al.. 2013. Deciphering the cryptic genome: genome-wide analyses of the rice pathogen Fusarium fujikuroi reveal complex regulation of secondary metabolism and novel metabolites. PLoS Pathog 9:e1003475. doi:10.1371/journal.ppat.1003475 23825955 PMC3694855

[165] Connolly LR, Smith KM, Freitag M. 2013. The Fusarium graminearum histone H3 K27 methyltransferase KMT6 regulates development and expression of secondary metabolite gene clusters. PLoS Genet 9:e1003916. doi:10.1371/journal.pgen.1003916 24204317 PMC3814326

[166] Som NF, Heine D, Holmes N, Knowles F, Chandra G, Seipke RF, Hoskisson PA, Wilkinson B, Hutchings MI. 2017. The MtrAB two-component system controls antibiotic production in Streptomyces coelicolor A3(2). Microbiology (Reading) 163:1415–1419. doi:10.1099/mic.0.000524 28884676 PMC5845573

[167] Muzio FM, Agaras BC, Masi M, Tuzi A, Evidente A, Valverde C. 2020. 7-hydroxytropolone is the main metabolite responsible for the fungal antagonism of Pseudomonas donghuensis strain SVBP6. Environ Microbiol 22:2550–2563. doi:10.1111/1462-2920.14925 31984618

[168] Cordero OX, Wildschutte H, Kirkup B, Proehl S, Ngo L, Hussain F, Le Roux F, Mincer T, Polz MF. 2012. Ecological populations of bacteria act as socially cohesive units of antibiotic production and resistance. Science 337:1228–1231. doi:10.1126/science.1219385 22955834

[169] Covington BC, Seyedsayamdost MR. 2022. Guidelines for metabolomics-guided transposon mutagenesis for microbial natural product discovery. Methods Enzymol 665:305–323. doi:10.1016/bs.mie.2021.11.020 35379440 PMC9078877

[170] Cain AK, Barquist L, Goodman AL, Paulsen IT, Parkhill J, van Opijnen T. 2020. A decade of advances in transposon-insertion sequencing. Nat Rev Genet 21:526–540. doi:10.1038/s41576-020-0244-x 32533119 PMC7291929

[171] Kwon YM, Ricke SC, Mandal RK. 2016. Transposon sequencing: methods and expanding applications. Appl Microbiol Biotechnol 100:31–43. doi:10.1007/s00253-015-7037-8 26476650

[172] van Opijnen T, Bodi KL, Camilli A. 2009. Tn-seq: high-throughput parallel sequencing for fitness and genetic interaction studies in microorganisms. Nat Methods 6:767–772. doi:10.1038/nmeth.1377 19767758 PMC2957483

[173] DeJesus MA, Nambi S, Smith CM, Baker RE, Sassetti CM, Ioerger TR. 2017. Statistical analysis of genetic interactions in Tn-Seq data. Nucleic Acids Res 45:e93. doi:10.1093/nar/gkx128 28334803 PMC5499643

[174] Goren MB, Brokl O, Schaefer WB. 1974. Lipids of putative relevance to virulence in Mycobacterium tuberculosis: phthiocerol dimycocerosate and the attenuation indicator lipid. Infect Immun 9:150–158. doi:10.1128/iai.9.1.150-158.1974 4271720 PMC414779

[175] Onwueme KC, Vos CJ, Zurita J, Ferreras JA, Quadri LEN. 2005. The dimycocerosate ester polyketide virulence factors of mycobacteria. Prog Lipid Res 44:259–302. doi:10.1016/j.plipres.2005.07.001 16115688

[176] Lerner TR, Queval CJ, Fearns A, Repnik U, Griffiths G, Gutierrez MG. 2018. Phthiocerol dimycocerosates promote access to the cytosol and intracellular burden of Mycobacterium tuberculosis in lymphatic endothelial cells. BMC Biol 16:1. doi:10.1186/s12915-017-0471-6 29325545 PMC5795283

[177] Wetmore KM, Price MN, Waters RJ, Lamson JS, He J, Hoover CA, Blow MJ, Bristow J, Butland G, Arkin AP, Deutschbauer A. 2015. Rapid quantification of mutant fitness in diverse bacteria by sequencing randomly bar-coded transposons. mBio 6:e00306-15. doi:10.1128/mBio.00306-15 25968644 PMC4436071

[178] Santiago M, Lee W, Fayad AA, Coe KA, Rajagopal M, Do T, Hennessen F, Srisuknimit V, Müller R, Meredith TC, Walker S. 2018. Genome-wide mutant profiling predicts the mechanism of a Lipid II binding antibiotic. Nat Chem Biol 14:601–608. doi:10.1038/s41589-018-0041-4 29662210 PMC5964011

[179] Schubert B, Maddamsetti R, Nyman J, Farhat MR, Marks DS. 2019. Genome-wide discovery of epistatic loci affecting antibiotic resistance in Neisseria gonorrhoeae using evolutionary couplings. Nat Microbiol 4:328–338. doi:10.1038/s41564-018-0309-1 30510172 PMC6663919

[180] Pensar J, Puranen S, Arnold B, MacAlasdair N, Kuronen J, Tonkin-Hill G, Pesonen M, Xu Y, Sipola A, Sánchez-Busó L, Lees JA, Chewapreecha C, Bentley SD, Harris SR, Parkhill J, Croucher NJ, Corander J. 2019. Genome-wide epistasis and co-selection study using mutual information. Nucleic Acids Res 47:e112–e112. doi:10.1093/nar/gkz656 31361894 PMC6765119

[181] Li YF, Costello JC, Holloway AK, Hahn MW. 2008. “Reverse ecology” and the power of population genomics. Evolution 62:2984–2994. doi:10.1111/j.1558-5646.2008.00486.x 18752601 PMC2626434

[182] Shapiro BJ. 2014. Signatures of natural selection and ecological differentiation in microbial genomes. Adv Exp Med Biol 781:339–359. doi:10.1007/978-94-007-7347-9_17 24277308

[183] Foster PL. 2004. Adaptive mutation in Escherichia coli. J Bacteriol 186:4846–4852. doi:10.1128/JB.186.15.4846-4852.2004 15262917 PMC451643

[184] Rocha EPC. 2018. Neutral theory, microbial practice: challenges in bacterial population genetics. Mol Biol Evol 35:1338–1347. doi:10.1093/molbev/msy078 29684183

[185] Azarian T, Huang I-T, Hanage WP. 2020. Structure and dynamics of bacterial populations: pangenome ecology. In Tettelin H, Medini D (ed), The pangenome: diversity, dynamics and evolution of genomes. Springer, Cham, Switzerland. 32633912

[186] Koskella B, Vos M. 2015. Adaptation in natural microbial populations. Annu Rev Ecol Evol Syst 46:503–522. doi:10.1146/annurev-ecolsys-112414-054458

[187] Shapiro BJ, Friedman J, Cordero OX, Preheim SP, Timberlake SC, Szabó G, Polz MF, Alm EJ. 2012. Population genomics of early events in the ecological differentiation of bacteria. Science 336:48–51. doi:10.1126/science.1218198 22491847 PMC3337212

[188] Bendall ML, Stevens SL, Chan L-K, Malfatti S, Schwientek P, Tremblay J, Schackwitz W, Martin J, Pati A, Bushnell B, Froula J, Kang D, Tringe SG, Bertilsson S, Moran MA, Shade A, Newton RJ, McMahon KD, Malmstrom RR. 2016. Genome-wide selective sweeps and gene-specific sweeps in natural bacterial populations. ISME J 10:1589–1601. doi:10.1038/ismej.2015.241 26744812 PMC4918448

[189] Zhao S, Lieberman TD, Poyet M, Kauffman KM, Gibbons SM, Groussin M, Xavier RJ, Alm EJ. 2019. Adaptive evolution within gut microbiomes of healthy people. Cell Host Microbe 25:656–667. doi:10.1016/j.chom.2019.03.007 31028005 PMC6749991

[190] Shapiro BJ. 2017. The population genetics of pangenomes. Nat Microbiol 2:1574. doi:10.1038/s41564-017-0066-6 29176697

[191] Li Y, Pinto-Tomás AA, Rong X, Cheng K, Liu M, Huang Y. 2019. Population genomics insights into adaptive evolution and ecological differentiation in streptomycetes. Appl Environ Microbiol 85:e02555-18. doi:10.1128/AEM.02555-18 30658977 PMC6585496

[192] Wang J, Li Y, Pinto-Tomás AA, Cheng K, Huang Y. 2022. Habitat adaptation drives speciation of a Streptomyces species with distinct habitats and disparate geographic origins. mBio 13:e02781-21. doi:10.1128/mbio.02781-21 35012331 PMC8749437

[193] Wyatt MA, Wang W, Roux CM, Beasley FC, Heinrichs DE, Dunman PM, Magarvey NA. 2010. Staphylococcus aureus nonribosomal peptide secondary metabolites regulate virulence. Science 329:294–296. doi:10.1126/science.1188888 20522739

[194] Spagnolo F, Trujillo M, Dennehy JJ. 2021. Why do antibiotics exist? mBio 12:e01966-21. doi:10.1128/mBio.01966-21 34872345 PMC8649755

[195] Stubbendieck RM, May DS, Chevrette MG, Temkin MI, Wendt-Pienkowski E, Cagnazzo J, Carlson CM, Gern JE, Currie CR. 2019. Competition among nasal bacteria suggests a role for siderophore-mediated interactions in shaping the human nasal microbiota. Appl Environ Microbiol 85:e02406-18. doi:10.1128/AEM.02406-18 30578265 PMC6498180

[196] Van Goethem MW, Osborn AR, Bowen BP, Andeer PF, Swenson TL, Clum A, Riley R, He G, Koriabine M, Sandor L, Yan M, Daum CG, Yoshinaga Y, Makhalanyane TP, Garcia-Pichel F, Visel A, Pennacchio LA, O’Malley RC, Northen TR. 2021. Long-read metagenomics of soil communities reveals phylum-specific secondary metabolite dynamics. Commun Biol 4:1302. doi:10.1038/s42003-021-02809-4 34795375 PMC8602731

[197] Medema MH, Cimermancic P, Sali A, Takano E, Fischbach MA. 2014. A systematic computational analysis of biosynthetic gene cluster evolution: lessons for engineering biosynthesis. PLoS Comput Biol 10:e1004016. doi:10.1371/journal.pcbi.1004016 25474254 PMC4256081

[198] Cruz-Morales P, Kopp JF, Martínez-Guerrero C, Yáñez-Guerra LA, Selem-Mojica N, Ramos-Aboites H, Feldmann J, Barona-Gómez F. 2016. Phylogenomic analysis of natural products biosynthetic gene clusters allows discovery of arseno-organic metabolites in model streptomycetes. Genome Biol Evol 8:1906–1916. doi:10.1093/gbe/evw125 27289100 PMC4943196

[199] Waglechner N, McArthur AG, Wright GD. 2019. Phylogenetic reconciliation reveals the natural history of glycopeptide antibiotic biosynthesis and resistance. Nat Microbiol 4:1862–1871. doi:10.1038/s41564-019-0531-5 31406334

[200] Sélem-Mojica N, Aguilar C, Gutiérrez-García K, Martínez-Guerrero CE, Barona-Gómez F. 2019. EvoMining reveals the origin and fate of natural product biosynthetic enzymes. Microb Genom 5:e000260. doi:10.1099/mgen.0.000260 30946645 PMC6939163

[201] Jensen RA. 1976. Enzyme recruitment in evolution of new function. Annu Rev Microbiol 30:409–425. doi:10.1146/annurev.mi.30.100176.002205 791073

[202] Booth TJ, Bozhüyük KAJ, Liston JD, Batey SFD, Lacey E, Wilkinson B. 2022. Bifurcation drives the evolution of assembly-line biosynthesis. Nat Commun 13:3498. doi:10.1038/s41467-022-30950-z 35715397 PMC9205934

[203] Nivina A, Herrera Paredes S, Fraser HB, Khosla C. 2021. GRINS: genetic elements that recode assembly-line polyketide synthases and accelerate their diversification. Proc Natl Acad Sci USA 118. doi:10.1073/pnas.2100751118 PMC8256042 34162709

[204] Brandis G, Hughes D. 2020. The SNAP hypothesis: chromosomal rearrangements could emerge from positive selection during niche adaptation. PLoS Genet 16:e1008615. doi:10.1371/journal.pgen.1008615 32130223 PMC7055797

[205] Medema MH, Trefzer A, Kovalchuk A, van den Berg M, Müller U, Heijne W, Wu L, Alam MT, Ronning CM, Nierman WC, Bovenberg RAL, Breitling R, Takano E. 2010. The sequence of a 1.8-mb bacterial linear plasmid reveals a rich evolutionary reservoir of secondary metabolic pathways. Genome Biol Evol 2:212–224. doi:10.1093/gbe/evq013 20624727 PMC2997539

[206] Rankin DJ, Rocha EPC, Brown SP. 2011. What traits are carried on mobile genetic elements, and why? Heredity (Edinb) 106:1–10. doi:10.1038/hdy.2010.24 20332804 PMC3183850

[207] Van Arnam EB, Ruzzini AC, Sit CS, Horn H, Pinto-Tomás AA, Currie CR, Clardy J. 2016. Selvamicin, an atypical antifungal polyene from two alternative genomic contexts. Proc Natl Acad Sci U S A 113:12940–12945. doi:10.1073/pnas.1613285113 27803316 PMC5135293

[208] Dragoš A, Andersen AJC, Lozano-Andrade CN, Kempen PJ, Kovács ÁT, Strube ML. 2021. Phages carry interbacterial weapons encoded by biosynthetic gene clusters. Curr Biol 31:3479–3489. doi:10.1016/j.cub.2021.05.046 34186025

[209] Gluck-Thaler E, Ralston T, Konkel Z, Ocampos CG, Ganeshan VD, Dorrance AE, Niblack TL, Wood CW, Slot JC, Lopez-Nicora HD, Vogan AA. 2022. Giant starship elements mobilize accessory genes in fungal genomes. Mol Biol Evol 39:msac109. doi:10.1093/molbev/msac109 35588244 PMC9156397

[210] Saati-Santamaría Z. 2023. Global map of specialized metabolites encoded in prokaryotic plasmids. Microbiol Spectr 11:e01523-23. doi:10.1128/spectrum.01523-23 37310275 PMC10434180

[211] Choulet F, Aigle B, Gallois A, Mangenot S, Gerbaud C, Truong C, Francou F-X, Fourrier C, Guérineau M, Decaris B, Barbe V, Pernodet J-L, Leblond P. 2006. Evolution of the terminal regions of the Streptomyces linear chromosome. Mol Biol Evol 23:2361–2369. doi:10.1093/molbev/msl108 16956972

[212] Tidjani A-R, Lorenzi J-N, Toussaint M, van Dijk E, Naquin D, Lespinet O, Bontemps C, Leblond P. 2019. Massive gene flux drives genome diversity between sympatric Streptomyces conspecifics. mBio 10:e01533-19. doi:10.1128/mBio.01533-19 31481382 PMC6722414

[213] Dimitriu T, Misevic D, Lotton C, Brown SP, Lindner AB, Taddei F. 2016. Indirect fitness benefits enable the spread of host genes promoting costly transfer of beneficial plasmids. PLoS Biol 14:e1002478. doi:10.1371/journal.pbio.1002478 27270455 PMC4896427

[214] Doroghazi JR, Buckley DH. 2010. Widespread homologous recombination within and between Streptomyces species. ISME J 4:1136–1143. doi:10.1038/ismej.2010.45 20393569

[215] Horinouchi S, Beppu T. 2007. Hormonal control by A-factor of morphological development and secondary metabolism in Streptomyces. Proc Jpn Acad Ser B Phys Biol Sci 83:277–295. doi:10.2183/pjab/83.277 PMC3859367 24367152

[216] Behruznia M, Marin M, Farhat M, Thomas JC, Domingo-Sananes MR, Meehan CJ. 2024. The Mycobacterium tuberculosis complex pangenome is small and driven by sub-lineage-specific regions of difference. bioRxiv. doi:10.7554/eLife.97870.1 PMC12413193 40910469

[217] Mortimer TD, Weber AM, Pepperell CS. 2018. Signatures of selection at drug resistance loci in Mycobacterium tuberculosis. mSystems 3:e00108-17. doi:10.1128/mSystems.00108-17 29404424 PMC5790871

[218] Tajima F. 1989. Statistical method for testing the neutral mutation hypothesis by DNA polymorphism. Genetics 123:585–595. doi:10.1093/genetics/123.3.585 2513255 PMC1203831

[219] Yu J. 2012. Current understanding on aflatoxin biosynthesis and future perspective in reducing aflatoxin contamination. Toxins (Basel) 4:1024–1057. doi:10.3390/toxins4111024 23202305 PMC3509697

[220] Chang P-K, Cary JW, Yu J, Bhatnagar D, Cleveland TE. 1995. The Aspergillus parasiticus polyketide synthase gene pksA, a homolog of Aspergillus nidulans wA, is required for aflatoxin B1 biosynthesis. Mol Gen Genet 248:270–277. doi:10.1007/BF02191593 7565588

[221] Carbone I, Jakobek JL, Ramirez-Prado JH, Horn BW. 2007. Recombination, balancing selection and adaptive evolution in the aflatoxin gene cluster of Aspergillus parasiticus. Mol Ecol 16:4401–4417. doi:10.1111/j.1365-294X.2007.03464.x 17725568

[222] Drott MT, Lazzaro BP, Brown DL, Carbone I, Milgroom MG. 2017. Balancing selection for aflatoxin in Aspergillus flavus is maintained through interference competition with, and fungivory by insects. Proc Biol Sci 284:20172408. doi:10.1098/rspb.2017.2408 29263278 PMC5745424

[223] Drott MT, Debenport T, Higgins SA, Buckley DH, Milgroom MG. 2019. Fitness cost of aflatoxin production in Aspergillus flavus when competing with soil microbes could maintain balancing selection. mBio 10:e02782-18. doi:10.1128/mBio.02782-18 30782658 PMC6381279

[224] Salamzade R, Tran PQ, Martin C, Manson AL, Gilmore MS, Earl AM, Anantharaman K, Kalan LR. 2024. Zol & fai: large-scale targeted detection and evolutionary investigation of gene clusters. bioRxiv. doi:10.1101/2023.06.07.544063 PMC11795205 39907107

[225] Demain AL. 2014. Importance of microbial natural products and the need to revitalize their discovery. J Ind Microbiol Biotechnol 41:185–201. doi:10.1007/s10295-013-1325-z 23990168

[226] Ziemert N, Podell S, Penn K, Badger JH, Allen E, Jensen PR. 2012. The natural product domain seeker NaPDoS: a phylogeny based bioinformatic tool to classify secondary metabolite gene diversity. PLoS One 7:e34064. doi:10.1371/journal.pone.0034064 22479523 PMC3315503

[227] Parks DH, Chuvochina M, Rinke C, Mussig AJ, Chaumeil P-A, Hugenholtz P. 2022. GTDB: an ongoing census of bacterial and archaeal diversity through a phylogenetically consistent, rank normalized and complete genome-based taxonomy. Nucleic Acids Res 50:D785–D794. doi:10.1093/nar/gkab776 34520557 PMC8728215

[228] Stubbendieck RM, Vargas-Bautista C, Straight PD. 2016. Bacterial communities: interactions to scale. Front Microbiol 7:1234. doi:10.3389/fmicb.2016.01234 27551280 PMC4976088

[229] Donia MS, Cimermancic P, Schulze CJ, Wieland Brown LC, Martin J, Mitreva M, Clardy J, Linington RG, Fischbach MA. 2014. A systematic analysis of biosynthetic gene clusters in the human microbiome reveals A common family of antibiotics. Cell 158:1402–1414. doi:10.1016/j.cell.2014.08.032 25215495 PMC4164201

[230] Sugimoto Y, Camacho FR, Wang S, Chankhamjon P, Odabas A, Biswas A, Jeffrey PD, Donia MS. 2019. A metagenomic strategy for harnessing the chemical repertoire of the human microbiome. Science 366:eaax9176. doi:10.1126/science.aax9176 31582523 PMC12168155

[231] Libis V, Antonovsky N, Zhang M, Shang Z, Montiel D, Maniko J, Ternei MA, Calle PY, Lemetre C, Owen JG, Brady SF. 2019. Uncovering the biosynthetic potential of rare metagenomic DNA using co-occurrence network analysis of targeted sequences. Nat Commun 10:3848. doi:10.1038/s41467-019-11658-z 31451725 PMC6710260

[232] Pereira-Flores E, Medema M, Buttigieg PL, Meinicke P, Glöckner FO, Fernández-Guerra A. 2021. Mining metagenomes for natural product biosynthetic gene clusters: unlocking new potential with ultrafast techniques. bioRxiv. doi:10.1101/2021.01.20.427441

[233] Libis V, MacIntyre LW, Mehmood R, Guerrero L, Ternei MA, Antonovsky N, Burian J, Wang Z, Brady SF. 2022. Multiplexed mobilization and expression of biosynthetic gene clusters. Nat Commun 13:5256. doi:10.1038/s41467-022-32858-0 36068239 PMC9448795

[234] Klapper M, Hübner A, Ibrahim A, Wasmuth I, Borry M, Haensch VG, Zhang S, Al-Jammal WK, Suma H, Fellows Yates JA, Frangenberg J, Velsko IM, Chowdhury S, Herbst R, Bratovanov EV, Dahse H-M, Horch T, Hertweck C, González Morales MR, Straus LG, Vilotijevic I, Warinner C, Stallforth P. 2023. Natural products from reconstructed bacterial genomes of the Middle and Upper Paleolithic. Science 380:619–624. doi:10.1126/science.adf5300 37141315

[235] Loureiro C, Galani A, Gavriilidou A, Chaib de Mares M, van der Oost J, Medema MH, Sipkema D. 2022. Comparative metagenomic analysis of biosynthetic diversity across sponge microbiomes highlights metabolic novelty, conservation, and diversification. mSystems 7:e00357-22. doi:10.1128/msystems.00357-22 35862823 PMC9426513

[236] Chen L-X, Anantharaman K, Shaiber A, Eren AM, Banfield JF. 2020. Accurate and complete genomes from metagenomes. Genome Res 30:315–333. doi:10.1101/gr.258640.119 32188701 PMC7111523

[237] Bickhart DM, Kolmogorov M, Tseng E, Portik DM, Korobeynikov A, Tolstoganov I, Uritskiy G, Liachko I, Sullivan ST, Shin SB, Zorea A, Andreu VP, Panke-Buisse K, Medema MH, Mizrahi I, Pevzner PA, Smith TPL. 2022. Generating lineage-resolved, complete metagenome-assembled genomes from complex microbial communities. Nat Biotechnol 40:711–719. doi:10.1038/s41587-021-01130-z 34980911

[238] Bertrand D, Shaw J, Kalathiyappan M, Ng AHQ, Kumar MS, Li C, Dvornicic M, Soldo JP, Koh JY, Tong C, Ng OT, Barkham T, Young B, Marimuthu K, Chng KR, Sikic M, Nagarajan N. 2019. Hybrid metagenomic assembly enables high-resolution analysis of resistance determinants and mobile elements in human microbiomes. Nat Biotechnol 37:937–944. doi:10.1038/s41587-019-0191-2 31359005

[239] Nayfach S, Shi ZJ, Seshadri R, Pollard KS, Kyrpides NC. 2019. New insights from uncultivated genomes of the global human gut microbiome. Nature New Biol 568:505–510. doi:10.1038/s41586-019-1058-x PMC6784871 30867587

[240] Garud NR, Good BH, Hallatschek O, Pollard KS. 2019. Evolutionary dynamics of bacteria in the gut microbiome within and across hosts. PLoS Biol 17:e3000102. doi:10.1371/journal.pbio.3000102 30673701 PMC6361464

[241] Olm MR, Crits-Christoph A, Bouma-Gregson K, Firek BA, Morowitz MJ, Banfield JF. 2021. inStrain profiles population microdiversity from metagenomic data and sensitively detects shared microbial strains. Nat Biotechnol 39:727–736. doi:10.1038/s41587-020-00797-0 33462508 PMC9223867

[242] Gregory AC, Gerhardt K, Zhong Z-P, Bolduc B, Temperton B, Konstantinidis KT, Sullivan MB. 2022. MetaPop: a pipeline for macro- and microdiversity analyses and visualization of microbial and viral metagenome-derived populations. Microbiome 10:49. doi:10.1186/s40168-022-01231-0 35287721 PMC8922842

[243] Lieberman TD. 2022. Detecting bacterial adaptation within individual microbiomes. Phil Trans R Soc B 377:20210243. doi:10.1098/rstb.2021.0243 35989602 PMC9393564

[244] Yaffe E, Dethlefsen L, Patankar AV, Gui C, Holmes S, Relman DA. 2023. A short course of antibiotics selects for persistent resistance in the human gut. Research Square. doi:10.21203/rs.3.rs-3399159/v1

[245] Mabesoone MFJ, Leopold-Messer S, Minas HA, Chepkirui C, Chawengrum P, Reiter S, Meoded RA, Wolf S, Genz F, Magnus N, Piechulla B, Walker AS, Piel J. 2024. Evolution-guided engineering of trans-acyltransferase polyketide synthases. Science 383:1312–1317. doi:10.1126/science.adj7621 38513027 PMC11260071

[246] Scheuerl T, Hopkins M, Nowell RW, Rivett DW, Barraclough TG, Bell T. 2020. Bacterial adaptation is constrained in complex communities. Nat Commun 11:754. doi:10.1038/s41467-020-14570-z 32029713 PMC7005322

[247] Shade A, Peter H, Allison SD, Baho DL, Berga M, Bürgmann H, Huber DH, Langenheder S, Lennon JT, Martiny JBH, Matulich KL, Schmidt TM, Handelsman J. 2012. Fundamentals of microbial community resistance and resilience. Front Microbiol 3:417. doi:10.3389/fmicb.2012.00417 23267351 PMC3525951

[248] Costello EK, Stagaman K, Dethlefsen L, Bohannan BJM, Relman DA. 2012. The application of ecological theory toward an understanding of the human microbiome. Science 336:1255–1262. doi:10.1126/science.1224203 22674335 PMC4208626

